# Diagnostic Performance of Prostate Cancer Disease‐Specific Phenotypes Identified Using Real‐World Databases: A Systematic Review

**DOI:** 10.1002/pds.70236

**Published:** 2025-10-15

**Authors:** Ami Vyas, Shweta Kamat, Sadie Thomas, Connor Gambino, Britny R. Brown, Amit D. Raval

**Affiliations:** ^1^ College of Pharmacy, Department of Pharmacy Practice and Clinical Research University of Rhode Island Kingston Rhode Island USA; ^2^ Bayer Healthcare Pharmaceuticals Whippany New Jersey USA

**Keywords:** biochemical recurrence, metastasis, prostate cancer, real‐world databases, systematic review

## Abstract

**Background:**

Research using real‐world databases (RWD) often requires the development of computable phenotypes based on clinical reasoning‐based algorithms or prediction models with validation through a reference standard such as chart review. While there are studies reporting different phenotypes for key prostate cancer (PC) disease or outcomes, these have not been summarized systematically.

**Objectives:**

To conduct a systematic review (SR) to summarize validation statistics on PC‐specific phenotypes, including metastasis, biochemical recurrence (BCR), castration‐resistant prostate cancer (CRPC), hormone‐sensitive prostate cancer (HSPC), progression‐free survival, and performance status.

**Methods:**

We conducted a SR in accordance with the Preferred Reporting Items for Systematic Reviews and Meta‐Analysis of Diagnostic Test Accuracy Studies guidelines. We systematically searched PubMed/Medline and EMBASE for studies reporting algorithms and prediction models for PC phenotypes based on structured RWD published between 2012 and 2024. A summary of algorithms and prediction models, along with their respective estimates of diagnostic accuracy compared to reference standards and/or measures of uncertainty, was provided. An area under the curve (AUC) > 0.7 was considered an acceptable phenotype.

**Results:**

Out of 7427 retrieved citations, 29 unique retrospective studies (31 citations) were included. Both claims‐based codes and prediction model‐based classification for any metastasis and bone metastases had an acceptable performance with high AUC (0.88 and > 0.7, respectively) and high specificity (above 90%) with a few having moderate sensitivity (60% to 100%). The prediction model‐based BCR classification had acceptable performance (AUC > 0.7); however, claims‐based BCR had moderate performance statistics with sensitivity in the range of 3%–19% and specificity in the range of 83%–98%. One claims‐based algorithm for metastatic CRPC had high sensitivity (77%) and specificity (100%). Studies for mHSPC were based on clinical reasoning without assessing their diagnostic accuracy. Claims‐based algorithms for performance status had at least 75% sensitivity and relatively high specificity.

**Conclusions:**

Our SR highlights the acceptable accuracy of computable phenotypes for PC, including (bone) metastasis, BCR, and performance status within RWD. Further validation studies are needed for RWD‐based phenotypes to account for changes in therapeutic options in PC.

**Summary**

- Information in real‐world databases cannot directly identify complex clinical constructs, especially in oncology.
- This is first comprehensive review that assessed the validity of algorithms for prostate cancer phenotypes in real‐world databases.
- We identified the acceptable diagnostic performance of several prostate cancer specific disease outcomes including metastasis, biochemical recurrence, and performance status, identified from the real‐world databases.
- Our study helps researchers who utilize large databases to identify patients with prostate cancer with specific outcomes or key characteristics from the real‐world databases.

## 1 Introduction

Real‐world databases (RWD) represent an enormous opportunity to conduct epidemiology and outcomes research studies. Administrative claims and electronic health/medical records (EMR) are the most common sources of RWD. However, claims/EMR are healthcare by‐products of transactional data generated for billing and reimbursement purposes, and hence, epidemiological research often encounters several challenges in accurately identifying complex clinical constructs using RWD. Much of the coded and structured information in RWD cannot directly identify complex clinical constructs, especially in oncology. Phenotypes are developed using clinical rationale or advanced machine‐learning or prediction models. However, it is essential to establish the validity of phenotypes compared to the gold standard measure in epidemiology research.

Prostate cancer (PC) is the most commonly occurring cancer in the United States (US) men [1] and the second most commonly occurring cancer in men worldwide [2]. Using RWD to identify the disease population across the continuum of PC care can be challenging. For example, most newly diagnosed cases present with localized stages managed through primary therapy of either active surveillance/watchful waiting, surgery (radical prostatectomy), or radiation therapy with or without adjuvant androgen deprivation therapy (ADT) [3]. Over time, men with localized PC may progress to biochemical recurrence (BCR) state, which is characterized by a rise in prostate‐specific antigen (PSA) level after primary therapy. Medical castration or surgical castration is one of the mainstay therapies for BCR. Many of those with BCR may stop responding to ADT (called castration‐resistant PC [CRPC]), or spread outside the prostate gland (metastases). Metastatic PC where the cancer spreads outside the prostate gland but still responds to ADT is considered metastatic hormone‐sensitive PC (mHSPC); conversely, a state where cancer cells spread outside the prostate gland and no longer respond to ADT is considered metastatic CRPC (mCRPC). RWD do not either have direct diagnosis codes to denote these disease states or codes that are not consistently utilized. Phenotypes could be developed using clinical reasoning‐based criteria/algorithm or a prediction model with a gold standard measure for such complex measures.

Several studies have been conducted to validate the criteria of assessing metastasis [4], BCR [5], CRPC [6], and functional status [7, 8] in patients with PC using RWD. However, studies on specific algorithms, criteria, and measures of PC‐specific disease population and outcomes in RWD have not been systematically summarized. We conducted a systematic review to summarize and report key validation statistics on phenotypes available for key characteristics of PC including metastasis, mCRPC, nmCRPC mHSPC, BCR, and functional status/performance status.

## 2 Methods

This systematic review was performed in accordance with the Preferred Reporting Items for Systematic Reviews and Meta‐Analysis of Diagnostic Test Accuracy (PRISMA‐DTA) Studies guidelines [9].

### 2.1 Inclusion Criteria for the Systematic Review

Studies eligible for inclusion were real‐world (RW) observational studies published in English. The PIRD (population, index test, reference test, and diagnosis of interest) criteria used to determine study eligibility identified studies for inclusion if they included patients with PC, had an index measure or an algorithm/model used to identify at least one of the relevant key PC characteristics or outcomes, and, if available, had a reference standard to compare with the index measure. Studies that reported the development and validation of machine‐learning methods or nomogram prediction models that did not have a reference standard were also eligible, as such studies could provide important RWD‐based predictive models used to identify patients with disease phenotypes. Some studies that assessed the validity of functional status/performance status included the entire cancer population. These studies were also considered eligible for inclusion due to the ease of application of claims‐based indicators of functional status/performance status in the PC population and the unavailability of studies specific to the PC population.

### 2.2 Systematic Literature Search

We conducted a systematic search of RW studies in EMBASE and PubMed using a combination of keywords for *prostate cancer, cancer, real‐world evidence*, and each diagnosis of interest, from 2012 through October 2023 (Table S1). References of included studies and relevant systematic reviews were also searched.

### 2.3 Study Selection, Data Extraction, and Study Quality Assessment

Two reviewers (A.V., S.K.) screened the titles and abstracts of studies independently to determine eligibility for the full‐text review and then examined full‐text reports. Disagreements were resolved through discussion and/or by the third reviewer (B.B.).

Two reviewers (S.T., C.G.) extracted data from the included studies, and the third reviewer (A.V.) performed a quality check and resolved discrepancies. Data on study characteristics, patient population, and each diagnosis of interest, including the measure type, specific index measure/algorithm, sensitivity, specificity, and predictive values, along with 95% confidence intervals, C‐statistic, and area under the curve (AUC) statistic were extracted.

One reviewer (A.V.) appraised the risk of bias and applicability of eligible studies using version 2 of the Quality Assessment of Diagnostic Accuracy Studies (QUADAS‐2) tool [10] as per the recommendation of the PRISMA 2020 guideline [11]. Two reviewers (S.T., C.G.) independently validated data by performing the quality check of data on the study quality assessment to ensure that decisions were not solely reliant on one reviewer and hence reduced the risk of error.

## 3 Results

**FIGURE 1:**
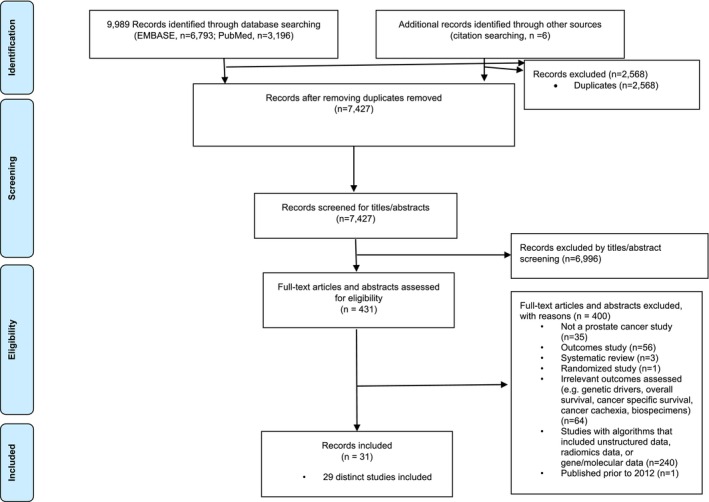
Preferred reporting items for systematic reviews and meta‐analysis (PRIMSA) flow diagram for study selection.

Out of 7427 retrieved records, 29 unique studies [4, 5, 6, 7, 8, 12, 13, 14, 15, 16, 17, 18, 19, 20, 21, 22, 23, 24, 25, 26, 27, 28, 29, 30, 31, 32, 33, 34, 35] (31 citations) met the inclusion criteria (Figure 1).

### 3.1 Study Characteristics and Patient Characteristics

All studies were retrospective studies, with several including US RWD (*n* = 18) (Table 1). Twenty‐three studies were full publications, while six studies were conference proceedings only. The patient sample size ranged from 212 to 1 144 610. Nineteen studies utilized at least an EMR or EMR linked to administrative claims data, while six studies used disease registry data, two studies used disease registry data linked to administrative claims data, and four studies used only administrative claims data.

**TABLE 1:** Characteristics of the included studies.

Phenotypes	Author, year of publication	RWD type	Source	Country	Study period	Cohort size
BCR	Abdul et al. (2019)^a^	EMR/Registry	Radiation Oncology Cancer Registry	Singapore	1998–2010	549
BCR	Hu et al. (2014)	EMR	NR	Germany	1999–2007	1575
BCR	Vincini et al. (2023)^a^	EMR	IEO European Institute of Oncology	Italy	2015–2018	949
BCR	Hassett et al. (2014)	Claims	HMO/CRN	USA	2000–2005	1151
BCR	Danciu et al. (2022)	EMR	VHA	USA	2002–2017	118 788
BCR, M1	Leapman et al. (2023)^a^	EMR‐Claims	Decipher genomic classifier‐Clarivate	USA	2013–2022	92 976
BCR, DM	Xiang et al. (2021)	EMR; Registry	iv. Multicountry EMR, ev. SEER and NCD	Multicountry	1995–2018	iv. 5275, ev. 23 989, 88 909
M1	Alba et al. (2021)	EMR	VHA	USA	2000–2020	1 144 610
M1	Dolan et al. (2012)	EMR	Robert Wood Johnson University Hospital	USA	1986–2007	292
M1	Nordstrom et al. (2012)	EMR‐Claims	EMR‐SDI Health	USA	2004–2010	267
M1	Preisser et al. (2020)	Registry	SEER	USA	2010–2014	201 224
M1	Shui et al. (2022)^a^	EMR	VHA	USA	2012–2017	722
M1	Thomsen et al. (2020)	Registry	Prostate Cancer Registry	Sweden	2006–2016	102 076
M1	Yang et al. (2022)	EMR	VHA	USA	NR	6211
M1, DM, BM	Ehrenstein et al. (2015)	Registry	Danish Registry	Denmark	2005–2010	212
BM	Bai et al. (2021)	EMR	NR	China	2014–2019	332
BM	Dong et al. (2022)	Registry‐Claims	SEER‐Medicare	USA	2010–2015	132 601
BM	Liu et al. (2021)	Registry; EMR	iv. SEER database, ev. Nanchung University EMR	USA, China	2010–2017	iv. 207 137, ev. 644
BM	Onukwugha et al. (2014)	Registry‐Claims	SEER‐Medicare	USA	2005–2007	2708
BM	Sathiakumar et al. (2017)	Claims	Medicare	USA	2005–2006	835
LNM	Jeong et al. (2012)	EMR	Seoul National University Hospital	Korea	1993–2009	2129
LNM	Sabbagh et al. (2023)	EMR	iv. University Medical Center Hamburg, ev. University of California‐San Francisco	USA, Germany	1990–2020	iv. 20 267, ev. 1322
mHSPC, mCRPC	Freedland et al. (2021)	Claims	Optum; Medicare	USA	2014–2019	21 675, 90 284
mCRPC	Thurin et al. (2021)	EMR	French Nationwide	France	2009–2014	386 127
mHSPC, mCRPC, nmHSPC, nmCRPC	Du et al. (2020)^a^	EMR, Claims	Optum	USA	2007–2018	125 505, 51 299
nmCRPC	Arnold et al. (2019)^a^	EMR	SAIL	Wales	2000–2015	38 021
nmCRPC	Malone et al. (2022)	Claims	Ontario Health Services, Alberta Health Services	Canada	2008–2019	866
PS/HS/DS	Davidoff et al. (2013)	Survey‐Claims	MCBS‐Medicare	USA	2001, 2003, 2005	14 788
PS	Sheffield et al. (2018)	EMR‐Claims	EMR‐MarketScan	USA	2007–2015	8442

Abbreviations: BCR, biochemical recurrence; BM, bone metastasis; DM, distant metastasis; DS, disability status; EMR, electronic medical records; HMO/CRN, health maintenance organization‐based cancer research network; HS, health status; LNM, lymph node metastasis; M1, metastasis; MCBS, medicare current beneficiary survey; mCRPC, metastatic castration‐resistant prostate cancer; mHSPC, metastatic hormone‐sensitive prostate cancer; NCD, national cancer database; nmCRPC, nonmetastatic castration‐resistant prostate cancer; nmHSPC, nonmetastatic hormone‐sensitive prostate cancer; NR, not reported; PS, performance status; SEER, surveillance, epidemiology, and end results; USA, United States of America; VHA, veterans health administration corporate data warehouse.

^a^ Abstracts/conference proceedings only.

Overall, the majority of studies included patients diagnosed with PC, with additional inclusion criteria used in several studies (Table S2). For instance, the Hu et al. study, which assessed the diagnostic accuracy of BCR, included patients with PC who underwent laparoscopic radical prostatectomy [21].

### 3.2 BCR

Seven studies assessed BCR phenotype [5, 12, 16, 21, 23, 33, 34] (Tables 2 and S3). Two studies that reported only ICD‐9/10 diagnosis code‐based definitions of BCR [5, 23] showed that such BCR definitions resulted in high specificity (98%) [5] or AUC value (96%) [23] when compared with the chart review or Decipher test, but low sensitivity (10%) and low positive predictive value (PPV) (31%) [5]. Hassett et al. added a criterion for chemotherapy initiation in addition to ICD‐9 codes, which led to an increase in sensitivity (19%); however, specificity (83%) and PPV (13%) declined when compared to the chart review [5].

**TABLE 2:** Diagnostic performance of biochemical recurrence phenotype.

Study ID	Condition (ICD‐9)	Condition (ICD‐10)	Medications	Procedures	Sensitivity % (95% CI)	Specificity % (95% CI)	PPV % (95% CI)	AUC % (95% CI)	Reference method
Claims‐based algorithms									
C									
Hassett (2014)	197–198.82	—	—	—	10 (5–19)^a^	98 (96–98)^a^	31^a^	—	CR
Leapman (2023)	—	R97.21	—	—	—	—	—	96^b^	Decipher test
C or M									
Hassett (2014)	197–198.82	—	Chemo	Chemo	19 (12–30)^a^	83 (80–86)^a^	11^a^	—	CR
M									
Hassett (2014)	—	—	Chemo	Chemo	3 (1–9)^a^	98 (97–99)^a^	13^a^	—	CR
Prediction‐based models									
	Type of prediction‐based model (predictors)								
Hu (2014)	(GS)					9 (7–11)^d^	—	72 (68–75)	PSA values
Hu (2014)	(PSA)					19 (17–21)^e^	—	62 (58–66)	PSA values
Hu (2014)	ANN (Age, PSA, % free PSA, prostate weight, DRE status, pathological stage, margin status, GS)				—	35 (33–38)^e^	—	75 (72–79)	PSA values
Hu (2014)	LR (Age, PSA, % free PSA, prostate weight, DRE status, pathological stage, margin status, GS)				—	37 (34–39)^e^	—	75 (72–79)	PSA values
Danciu (2022)	ML (Age, race, ethnicity, GS, AJCC stage group, SEER summary stage, computed stage value, PSA, the penultimate PSA over the last 5 years, and their rate of change)				—	—	—	76 (76–76)	NR
Vincini (2023)	GBDT (Age, comorbidities, risk class, PSA, T‐stage, N‐stage, preoperative GS and ISUP)				—	—	—	59	NR
Abdul (2019)^c^	NP (PSA, T‐stage, N‐stage, GS [primary, secondary, total])				—	—	—	—	NR
Xiang (2021)	NP (PSA, biopsy GGG, % positive cores, T‐stage)				—	—	—	63 (61–65)	CR

Abbreviations: AJCC, American Joint Committee on Cancer; ANN, artificial neural networks; AUC, area under the curve; C, claims; CI, confidence intervals; CR, chart review; DRE, digital rectal exam; GBDT, gradient boosted decision tree; GGG, Gleason Grade Group; GS, Gleason score; ICD, International Classification of Diseases; ISUP, International Society of Urological Pathology; LR, logistic regression; M, medications; ML, machine learning; NP, nomogram prediction; NR, not reported; PPV, positive predictive value; PSA, prostate specific antigen; SEER, surveillance, epidemiology and end results.

^a^ At 5 years follow‐up.

^b^ Concordance value.

^c^ 92% of patients with BCR at 5 years' follow‐up.

^d^ At 95% sensitivity.

^e^ At 90% sensitivity.

Five studies reported the diagnosed accuracy of model‐based prediction for BCR. One study used artificial neural network and logistic regression (LR) prediction models with age and tumor characteristics and reported a specificity of 35% at a sensitivity value of 90%, with AUCs of 75% for both models [21] when compared to PSA values reported in the data, while another study used a machine‐learning model with tumor characteristics and demographic factors and reported an AUC of 76% (95% CI: 76%–76%) [16]. Yet another study used a nomogram prediction model with tumor characteristics and reported an AUC of 63.0% (95% CI: 61.0%–65.0%) [34], while Vincini et al. used a Gradient‐boosted decision tree model with clinical variables and tumor characteristics and reported an AUC of 59.0% [33].

### 3.3 PC Metastasis

#### 3.3.1 Any Metastasis

Nine studies [4, 13, 17, 20, 23, 27, 30, 31, 35] assessed the metastasis phenotype (Tables 3 and S4). Four studies [13, 17, 30, 35] used only ICD‐9/10 diagnosis code‐based definitions of metastasis, which reported a high sensitivity in the range of 73% [30, 35] to 95% [17], a high specificity of 92% [30] to 100% [17], and also a high PPV of 78% [35] to 100% [17]. Alba et al. added a criterion for androgen receptor pathway inhibitors (ARPI) along with ICD‐9/10 codes, resulting in an increase in sensitivity from 81% to 90% but a slight decline in specificity from 95% to 92% [13]. Two studies that used a combination of ICD‐9/10 codes with ARPI, chemotherapy, and/or other drugs indicated for metastatic PC reported a sensitivity of approximately 80%, specificity in the range of 75% [4] and 89% [30], and PPV of 75% [30] to 86% [4]. Alba et al. also reported a sensitivity of 60%, specificity of 100%, and PPV of 78% for the algorithm of only ARPI use [13]. A study that combined ICD‐10 codes with PSA > 50 ng/mL with or without bone scintigraphy and bone health agents reported lower PPVs in the 16%–28% range [20]. All these studies used chart review or radiology reports as a reference standard for validation purposes.

**TABLE 3:** Diagnostic performance of metastases phenotypes.

Study ID	Condition (ICD‐9 or 10)	Medications	Laboratory	Procedures	Sensitivity % (95% CI)	Specificity % (95% CI)	PPV % (95% CI)	AUC % (95% CI)	Reference method
Any metastasis									
Claims‐based algorithms									
C									
Dolan (2012)	197.0, 197.7, 198.3, OR 198.5	—	—	—	95 (80–96)	100 (98–100)	100 (94–100)	—	CR
Dolan (2012)	198.5	—	—	—	90 (80–96)	100 (98–100)	100 (94–100)	—	CR
Alba (2021)	(198.1, 198.5, 196.5, 196.6) OR (C79.11, C79.51, C79.52, C77.4, C77.5)	—	—	—	81	95	88	—	CR
Yang (2022)	(196.x, 197.x, 198.x, 199.x) OR (C77.x, C78.x, C79.xx, C7B.0x, C7B.1, C7B.8)	—	—	—	73	86	78	—	RR
Shui (2022)	^a^	—	—	—	73 (66–79)	92 (89–94)	79^c^	—	CR
Leapman (2023)	^b^	—	—	—	NR	NR	NR	95^d^	Decipher test
C or M									
Alba (2021)	(198.1, 198.5, 196.5, 196.6) OR (C79.11, C79.51, C79.52, C77.4, C77.5)	ARPI	—	—	90	92	93	—	CR
Nordstrom (2012)	197.xx‐198.xx, 199.xx	ARPI, CHEMO, BHA	—	—	81	75	86	82	EMR
Shui (2022)	^a^	Metastatic PC drugs	—	—	80 (74–86)	89 (86–91)	75^c^	—	CR
M									
Alba (2021)	—	ARPI	—	—	60	100	78	—	CR
C + L									
Ehrenstein (2015)	C77‐C79	—	PSA > 50 ng/mL	—	NR	NR	16 (6–32)^e^	—	CR
C + L + *P*									
Ehrenstein (2015)	C77‐C79	—	PSA > 50 ng/mL	BSc	NR	NR	28 (14–47)^e^	—	CR
C + L+(M or P)									
Ehrenstein (2015)	C77‐C79	BHA	PSA > 50 ng/mL	BSc	NR	NR	15 (6–30)^e^	—	CR
Prediction‐based models									
	Type of prediction‐based model (predictors)								
Preisser (2020)	LR (GGG, PSA, tumor stage)				—	—	—	94 (94–94)	DR
Thomsen (2020)	Age, year, mode of detection, TNM, PSA, T‐stage, GGG, primary treatment, CCI, education, marital status				—	—	—	88	DR
Bone metastasis									
Claims‐based algorithms									
C									
Onukwugha (2014)	198.5	—	—	—	60 (57–62)	54 (51–57)	68 (66–71)	—	DR
C+(M or P)									
Onukwugha (2014)	198.5	BHA	—	BSc, BB	56 (53–58)	58 (55–61)	69 (67–72)	—	DR
C + *P*									
Sathiakumar (2017)	198.5	—	—	BM‐related procedures^h^	92 (78–97)	99 (96–100)	92 (78–97)	—	CR
C + L									
Ehrenstein (2015)	C77‐C79	—	PSA > 50 ng/mL	—	NR	NR	11 (3–25)^e^	—	CR
C + L + *P*									
Ehrenstein (2015)	C77‐C79	—	PSA > 50 ng/mL	BSc	NR	NR	9 (2–25)^e^	—	CR
C + L+(M or P)									
Ehrenstein (2015)	C77‐C79	BHA	PSA > 50 ng/mL	BSc	NR	NR	5 (1–17)^e^	—	CR
Prediction‐based models									
	Type of prediction‐based model (predictors)								
Bai (2021)	NP (Age, tumor stage, PSA, GS, prostate volume, red cell distribution width, SAP, neutrophil/lymphocyte %)				—	—	—	96 (93–98)	Bone Scan/MRI
Dong (2022)	NP (Age, race, marital status, grade, PSA, ISUP, T‐stage, N‐stage, brain/liver/lung metastasis)				—	—	—	95	DR
Liu (2021)	ML‐XGB (Age, race, grade, PSA, GS, T‐stage, N‐stage, marital status)				91^f^; 91^g^	88^f^; 88^g^	—	96^f^; 96^g^	DR
Liu (2021)	ML‐DT (Age, race, grade, PSA, GS, T‐stage, N‐stage, marital status)				88^f^; 85^g^	83^f^; 88^g^	—	94^f^; 94^g^	DR
Liu (2021)	ML‐RF (Age, race, grade, PSA, GS, T‐stage, N‐stage, marital status)				90^f^; 88^g^	88^f^; 87^g^	—	95^f^; 95^g^	DR
Liu (2021)	ML‐MLP (Age, race, grade, PSA, GS, T‐stage, N‐stage, marital status)				90^f^; 91^g^	88^f^; 87^g^	—	95^f^; 95^g^	DR
Liu (2021)	ML‐LR (Age, race, grade, PSA, GS, T‐stage, N‐stage, marital status)				87^f^; 87^g^	85^f^; 85^g^	—	90^f^; 91^g^	DR
Liu (2021)	ML‐NBC (Age, race, grade, PSA, GS, T‐stage, N‐stage, marital status)				89^f^; 91^g^	88^f^; 86^g^	—	94^f^; 93^g^	DR
Lymph node metastasis									
Prediction‐based models									
	Type of prediction‐based model (predictors)								
Jeong (2012)	NP (PSA, stage, GS, % positive cores)							84^f^; 97^g^	PA
Sabbagh (2023)	ML‐SLR (Age, PSA, T‐stage, % positive cores, primary and secondary GS)				—	—	—	81 (80–82)^f^; 81^g^	NR
Sabbagh (2023)	ML‐LRE (Age, PSA, T‐stage, % positive cores, primary and secondary GS)				—	—	—	81 (80–82)^f^; 81^g^	NR
Sabbagh (2023)	ML‐XGB (Age, PSA, T‐stage, % positive cores, primary and secondary GS)				—	—	—	82 (81–83)^f^; 82^g^	NR
Distant metastasis									
Claims‐based algorithms									
C + L									
Ehrenstein (2015)	C77‐C79	—	PSA > 50 ng/mL	—	NR	NR	5 (1–18)^e^	—	CR
C + L + *P*									
Ehrenstein (2015)	C77‐C79	—	PSA > 50 ng/mL	BSc	NR	NR	19 (7–36)^e^	—	CR
C + L+(M or P)									
Ehrenstein (2015)	C77‐C79	BHA	PSA > 50 ng/mL	BSc	NR	NR	10 (3–24)^e^	—	CR
Prediction‐based models									
	Type of prediction‐based model (predictors)								
Xiang (2021)	NP (PSA, GGG, % positive cores, T‐stage)				—	—	—	69 (66–71)	EMR

Abbreviations: ARPI, androgen receptor pathway inhibitors; AUC, area under the curve; BHA, bone health agent; BSc, bone scintigraphy/scan; C, claims; CCI, Charlson Comorbidity Index; CI, confidence intervals; CHEMO, chemotherapy; CR, chart review; DR, disease registry; DT, decision tree; EMR, electronic medical records; GGG, Gleason Grade Group; GS, Gleason score; ICD, International Classification of Diseases; ISUP, International Society for Urological Pathology; L, laboratory values; LR, logistic regression; LRE, logistic regression ensemble; M, medications; ML, machine learning; MLP, multilayer perceptron; NBC, naïve bayes classification; NP, nomogram prediction; NR, not reported; P, procedures; PA, pathological assessment; PC, prostate cancer; PPV, positive predictive value; PSA, prostate‐specific antigen; RF, random forest; RR, radiology reports; SAP, serum alkaline phosphatase; SEER, surveillance, epidemiology and end results; SLR, standard logistic regression; TNM, tumor node metastasis; XGB, extreme gradient boosting.

^a^ ICD‐9/10 codes for secondary malignant neoplasms.

^b^ Diagnosis codes for metastasis.

^c^ At metastatic prostate cancer prevalence of 30%.

^d^ Concordance value.

^e^ 180 days before or after the index date.

^f^ Internal validation.

^g^ External validation.

^h^ Included radiation therapy for bone metastasis, bone surgery, fracture other than spine or spine fracture, changes in chemotherapy, spinal cord compression, bone biopsy, bone scan, X‐rays of pelvis/femur, magnetic resonance imaging of back, positron emission tomography scan, bone pain, high alkaline phosphatase plus hypercalemia.

A study that used a LR model of tumor characteristics reported an AUC of 94% (95% CI: 94%–94%) [27], while another study that used a prediction model of tumor characteristics and demographic factors reported an AUC of 88% [31]. Both the studies that used prediction‐based models utilized data from the disease registry as the reference standard.

#### 3.3.2 Bone Metastasis

Six studies [15, 18, 20, 24, 26, 29] assessed the bone metastasis (BM) phenotype (Tables 3 and S4). Three studies reported only claims‐based definitions of BM [20, 26, 29]. One study with a combination of an ICD‐9 code and BM‐related procedures resulted in a sensitivity of 92% (95% CI: 78%–97%), a specificity of 99%, and PPV of 92% when compared with data from chart review [29], while another study that used an ICD‐9 code with or without bone scans and bone health agents when compared with data from the disease registry resulted in a sensitivity of 56%–60%, a specificity of 54%–58%, and PPV of around 69% [26]. Yet another study that used a combination of ICD‐10 codes with PSA > 50 ng/mL with or without bone scintigraphy and bone health agents reported lower PPVs in the 5%–11% range when compared with data from chart review [20].

Three studies reported the diagnostic accuracy of model‐based prediction for the BM phenotype using data from bone scans or the disease registry as the reference standard [15, 18, 24]. Bai et al. [15] and Dong et al. [18] used nomogram prediction models based on tumor characteristics and reported AUCs of 96% and 95%, respectively. While another study used machine‐learning models of tumor characteristics and demographic factors and described AUCs of 90% from the LR model and 96% from the eXtreme Gradient Boosting model, and sensitivity in the 87%–91% range when compared with the data from the disease registry [24].

#### 3.3.3 Lymph Node Metastasis

Two studies reported the diagnostic accuracy of model‐based prediction for LNM using data from hospital EHR (Tables 3 and S4). Jeong et al. used a nomogram prediction model of tumor characteristics and reported an AUC of 97% [22]. Sabbagh et al. used machine‐learning models of tumor characteristics and reported AUCs of 82.0% [28].

#### 3.3.4 Distant Metastasis

One study reported the diagnostic accuracy of model‐based prediction for distant metastasis phenotype (Tables 3 and S4) and reported an AUC of 69% [34], while another study that used a combination of ICD‐10 codes with PSA > 50 ng/mL with or without bone scintigraphy and bone health agents reported lower PPVs in the range of 5%–19% when compared with data from chart review [20].

### 3.4 Advanced PC Phenotypes

#### 3.4.1 CRPC

Three studies assessed the mCRPC phenotype [6, 19, 32], while three studies [14, 19, 25] assessed the nmCRPC phenotype (Tables 4 and S5). Thurin et al., reported only a claims‐based definition of mCRPC, which had a sensitivity of 77%, a specificity of 100%, and a PPV of 97% using data from chart review as a reference standard [32]. A study that used an algorithm of claims‐based codes and/or laboratory values reported that the algorithm identified approximately 12%–13% of patients with mCRPC from the databases they utilized [6]. Another study used an algorithm with a combination of claims‐based codes and laboratory values and identified < 5% of patients with mCRPC [19]. None of the latter two studies assessed the diagnostic accuracy of algorithms. Malone et al., assessed three separate algorithms for nmCRPC using a combination of claims for either surgical or medical castration along with PSA values and reported sensitivity in the 53%–57% range, specificity in the 68%–80% range, and PPV in the 28%–36% range using data from chart review as a reference standard [25], while Du et al., used claims‐based codes and laboratory values and identified 34% of patients with nmCRPC from the dataset used in the study [19].

**TABLE 4:** Diagnostic performance of advanced prostate cancer phenotypes.

Study ID	Criteria	Condition (ICD‐9 or 10)	Medications	Laboratory	Procedures	Sensitivity (95% CI)	Specificity (95% CI)	PPV (95% CI)	% with Outcomes	Reference method
Metastatic castration‐resistant prostate cancer										
Freedland (2021)	Criteria 1: C, **OR**	Z19.2	—	—	—	NR	NR	NR	Optum: 12%; Medicare: 13%	NR
	Criteria 2: *P* + L, **OR**	—	—	Rise in PSA after SC	SC^a^					
	Criteria 3: *P* + C, **OR**	Z19.2 after SC	—	—	SC^a^					
	Criteria 4: M + L, **OR**	—	MC (≥ 90 days of ADT)	Rise in PSA during MC	—					
	Criteria 5: M + C, **OR**	R97.21 during MC	MC (≥ 90 days of ADT)	—	—					
	Criteria 6: *P* + C, **OR**	First metastases post 90 days of SC	—	—	SC^a^					
	Criteria 7: M + C, **OR**	First metastases post 90 days of MC	MC (≥ 90 days of ADT)	—	—					
	Criteria 8: M	—	mCRPC medications only^b^	—	—					
Du (2020)	Criteria 1: (C + (M or P)) **AND**	PC diagnosis	MC	—	SC				Optum EMR: 3%; Optum claims: 2%	NR
	Criteria 2: (C or M or L)	Z19.2 post‐PC diagnosis	New ARPI initiation	Rise in PSA after SC/MC	—					
Thurin (2021)	Criteria 1: (C or M or P) **AND**	C77, C78, C79	(BHA) or (mCRPC prescription 90 days post‐ADT)	—	RT^c^	77%	100%	97% (93%–100%)	—	CR
	Criteria 2: (M or P)	—	ADT and CRPC/mCRPC prescriptions post 90 days of PC diagnosis	—	SC					
Nonmetastatic castration‐resistant prostate cancer										
Du (2020)	Criteria 1: (M or P) **AND**	—	MC	—	SC	—	—	—	Optum EMR: 10%; Optum claims: 34%	NR
	Criteria 2: (C or M or L)	Z19.2 post‐SC/MC	New ARPI Initiation post‐SC/MC	Rise in PSA after SC/MC	—					
Arnold (2019)	C or M or P or L	No metastases	ADT	PSA results	No metastases	—	—	—	4% (3%–4%)	NR
Malone (2022)	(M or P) + L	—	1 year of ADT use	PSA < 20 ng/mL within 90 days prior to ADT initiation	SC	53%	80%	36%	—	CR
Malone (2022)	M + L	—	1 year of ADT use	PSA < 20 ng/mL within 90 days prior to ADT initiation	—	61%	68%	28%	—	CR
Malone (2022)	(M or P) + L	—	ADT use	PSA < 20 ng/mL within 90 days prior to ADT initiation	SC	57%	70%	28%	—	CR
Metastatic hormone‐sensitive prostate cancer										
Freedland (2021)	Criteria 1: (C or M or L or P) **AND**	No evidence of CRes premetastasis^d^	No evidence of CRes premetastases^d^	No evidence of CRes premetastases^d^	No evidence of CRes premetastases^d^	—	—	—	Optum: 32%; Medicare: 22%	NR
	Criteria 2: C or (*P* + L) or (M + L)	Z19.1 within 12 months premetastasis	MC (≥ 90 days of ADT prior to metastasis)	≥ 2 PSA rise results following SC/MC and within 12 months premetastasis	SC^a^ prior to metastasis					
Du (2020)	C + M	First metastasis between PC and ADT	ADT use post‐PC diagnosis	—	—	—	—	—	Optum EMR: 1%; Optum claims: 2%	NR
Nonmetastatic hormone‐sensitive prostate cancer										
Du (2020)	C + M + L + *P*	No metastasis between PC and ADT	ADT use post‐PC	No rise in PSA	No metastasis	—	—	—	Optum EMR: 3%; Optum claims: 30%	NR

Abbreviations: ADT, androgen deprivation therapy; ARPI, androgen receptor pathway inhibitors; BHA, bone health agent; C, claims; CI, confidence intervals; CR, chart review; CRes, castration resistance; CRPC, castration‐resistant prostate cancer; EMR, electronic medical records; ICD, International Classification of Diseases; L, laboratory values; M, medications; MC, medical castration; mCRPC, metastatic castration‐resistant prostate cancer; mL, milliliters; ng, nanogram; NR, not reported; P, procedures; PC, prostate cancer; PPV, positive predictive value; PSA, prostate‐specific antigen; RT, radiation therapy; SC, surgical castration.

^a^ Bilateral orchiectomy, two unilateral orchiectomies.

^b^ Enzalutamide, diethyl stilbesterol, polyestrodiol phosphate, estramustine phosphate, pembrolizumab, sipuleucel‐T, radium‐223, etoposide, carboplatin, cisplatin, cabazitaxel, docetaxel, mitoxantrone.

^c^ Included nonintensity modulated radiation therapy, intensity modulated radiation therapy, or stereotactic radiotherapy.

^d^ Identified from the Freedland 2021 algorithm for mCRPC earlier in the table.

#### 3.4.2 HSPC

Two studies assessed the mHSPC phenotype [6, 19], while one study assessed the nmHSPC phenotype [19] (Tables 4 and S5). Freedland et al. used an algorithm that combined claims‐based codes and laboratory values and found that the algorithm identified 22%–32% of patients with mHSPC from the databases they utilized [6], while another study that used only claims‐based codes identified < 5% of patients with mHSPC [19]. Du et al. also used a combination of claims and laboratory values and identified 30% of patients with nmHSPC from the database they utilized [19]. None of these studies assessed the diagnostic accuracy of algorithms.

### 3.5 Performance Status

Two studies [7, 8] assessed the diagnostic accuracy of performance status using claims‐based predictors in patients with cancer (Table S6). Sensitivity was reported in both studies and was at least 75%, while specificity was 92% in one study [8] and 75% in another study [7]. PPV was 48% and negative predictive value was 98% in one study [8]. Both studies used data from self‐reports and/or patient‐reported outcomes as a reference standard.

### 3.6 Quality Assessment

**FIGURE 2:**
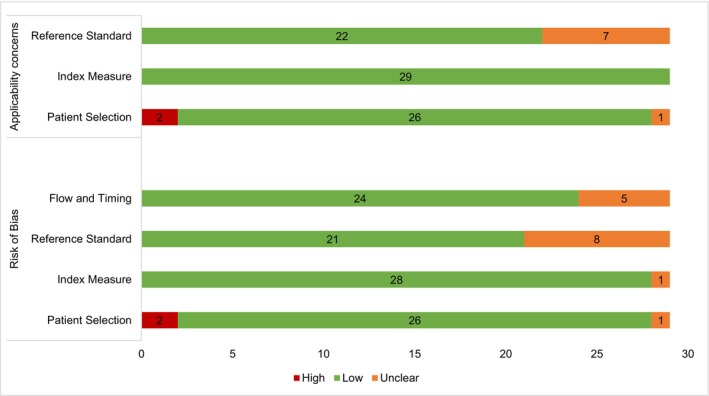
Study quality assessment using QUADAS‐2 tool. The red, green, and orange color indicates the level of risk of bias and applicability concerns as assessed using the QUADAS‐2 tool.

A total of 17 studies (58.6%) were deemed to be at low risk of bias on all domains and also of low concern with regard to applicability (Table S7), while two studies [7, 8] had a high risk of bias and applicability concerns with regard to patient selection due to their inclusion of the entire cancer patient populations (Figure 2).

## 4 Discussion

To the best of our knowledge, this is the first comprehensive systematic review that addressed the validity of algorithms for PC phenotypes in RWD from published and unpublished data. We identified several validation studies of PC key phenotypes, ranging from two studies that assessed performance status to nine studies that assessed any metastasis.

Our systematic review reported several noteworthy findings. Findings for BCR indicate that the prediction‐based models have AUC values < 75% [21, 33, 34], indicating that such models may not sufficiently identify all patients with BCR from RWD. However, ICD‐9 or ICD‐10‐based algorithms resulted in high specificity/AUC values, but very low sensitivity compared to data from chart reviews [5, 23]. Hence, there is an urgent need to develop and validate algorithms for identifying BCR with high sensitivity and specificity values from RWD.

Findings on any metastasis showed that the ICD‐9 or ICD‐10 codes for secondary neoplasm without [13, 30] or with other common metastatic cancer treatments [4, 30] in the US EMR data systems resulted in relatively high sensitivity and specificity when compared to data from chart review, thereby signaling that claims‐based algorithms could be used to identify patients with metastasis from RWD. However, as new metastatic agents become available and existing agents are incorporated into the guidelines to treat earlier stages of disease and as new disease classification systems become available (ICD‐9 to ICD‐10), these algorithms may need to be updated and/or revised and further validated. For instance, enzalutamide and darolutamide are now approved for the earlier stages of PC, and hence the algorithms of prescription of these medications for identifying patients with metastasis should not be used. The use of only ARPI to identify patients with any metastasis had lower validation parameters when compared to data from the chart review [13]. Hence, ICD codes should be used along with the codes to identify metastatic agents to accurately identify patients with PC metastasis. The combination of ICD‐10 codes and PSA value > 50 ng/mL with or without bone scintigraphy and bone health agents had low PPV values [20] indicating that such algorithms may not be helpful to accurately identify patients with metastasis from RWD. The lower PPV values in the later algorithms may be due to several reasons. First, the algorithms required a patient to meet both the criteria of ICD codes and PSA values. Higher PSA levels may precede the development of metastasis, and it is likely that not all components of the algorithm may be fulfilled when metastasis is assessed. Second, serum PSA levels alone can provide very little information about the presence of metastasis, especially in those with a new cancer diagnosis. We also found that studies that used prediction models [27, 31] had AUCs > 88% when compared to data from the disease registry as a reference standard, indicating that such models could be used to identify patients with metastasis in disease registries when claims‐based codes are not available.

Using only the secondary malignant neoplasm of bone and bone marrow 195.8 ICD‐9 code with or without procedure codes for bone scan, bone biopsy, and/or intravenous bisphosphonates resulted in relatively low sensitivity and specificity values compared to data from the disease registry [26], suggesting that such claims‐based algorithms may not sufficiently capture all patients with BM. However, using a combination of 198.5 ICD‐9 code with procedure codes for radiation therapy for BM, fracture, spine fracture, spine cord compression, or evaluation and management from the Medicare claims data [29], resulted in sensitivity, specificity, and PPV values > 92% compared to data from chart review as a reference standard, indicating that an appropriate combination of ICD‐9 codes with certain procedure codes could be utilized to identify patients with BM using administrative claims and EMR databases. The combination of ICD‐10 codes and PSA > 50 ng/mL with or without bone scintigraphy and bone health agents had very low PPV values when compared to the data from chart review, thereby suggesting that such algorithms may not be utilized to identify patients with BM from RWD. Future studies that validate algorithms using ICD‐10 codes along with bone‐metastasis related procedures are needed. Nomogram prediction models using tumor characteristics [15, 18] and machine‐learning models of tumor characteristics and demographic factors [24] to predict BM generated AUCs > 90% and sensitivity and specificity values of ~90%, respectively, when compared to data from the disease registry or bone scans, suggesting that such prediction models could be used to identify patients with BM from EMR and/or disease registries in the absence of claims‐based codes.

Prediction‐based models of LNM using tumor characteristics with or without demographic factors generated relatively high AUC values [22, 28] indicating the utility of such algorithms in the absence of direct measures from RWD. However, these predictive models were based on data from hospital EMR, suggesting limited generalizability. Such predictive models based on tumor characteristics may be used to identify patients with LNM from the disease registry databases and EMRs. Importantly, the models did not use a reference standard or an index test. Hence, future validation studies using a reference standard should be conducted. Our findings about the predictive ability of claims and laboratory values‐based algorithms [20] for distant metastasis indicate that such algorithms are not suitable for identifying patients with distant metastasis due to very low PPV. However, the predictability of distant metastasis from a nomogram model based on tumor characteristics was relatively higher [34]. Algorithms that help identify distant metastasis in patients with PC from RWD need to be developed and validated.

A French nationwide healthcare data study [32] that used claims‐based codes with or without laboratory values to identify patients with mCRPC had a relatively high sensitivity, specificity, and PPV when compared to data from the chart review, indicating that such an algorithm can sufficiently identify patients with mCRPC from RWD. However, medications for CRPC were also a component of the algorithm; hence, as new agents become available and/or guidelines for treating CRPC change, the algorithm may need to be updated, revised, and further validated. Freedland et al., used an algorithm with claims‐based codes with or without laboratory values to identify the proportion of patients with mCRPC [6] but did not assess its diagnostic accuracy. Similarly, Du et al. developed algorithms using a combination of claims‐based variables and laboratory values for mCRPC and nmCRPC but did not validate the algorithm [19]. Future studies that validate these algorithms in RWD are warranted. A study by Malone et al., has three different algorithms based on the combination of claims‐based codes and laboratory values to identify patients with nmCRPC [25]; however, sensitivity, specificity, and PPV were relatively low compared to the reference standard (chart review), and hence such algorithms could not sufficiently identify all patients with the disease phenotype from the EMR. Similarly, Freedland et al., used an algorithm with claims‐based codes with or without laboratory values to identify the proportion of patients with mHSPC [6]; however, they did not assess its diagnostic accuracy. Future studies assessing the validity of the algorithms for identifying patients with HSPC from RWD are warranted.

Our findings suggest that claims‐based algorithms for performance status have high specificity and relatively acceptable sensitivity, comparing such data with patient‐reported outcomes or self‐reports as a reference standard, indicating the utility of such algorithms for identifying patients with poor performance status, especially when such patient‐reported data is not directly available in EMR or administrative claims data.

This is the only comprehensive systematic review of the largest number of RW studies conducted in the last decade to summarize the diagnostic accuracy of algorithms for identifying PC‐specific phenotypes. Also, the overall quality of the studies included in the systematic review was assessed as good. There were some concerns about patient selection in the two studies that evaluated performance status due to their inclusion of the entire cancer patient population. However, this may not affect the findings as claims‐based algorithms for performance status can also be applied to PC patients following further validation. Moreover, several algorithms that utilized ICD‐9 codes with or without other claims‐based indicators and utilized data for years 2015 and earlier need to be updated and validated for ICD‐10 codes to ensure consistent performance. In our review, two studies that used ICD‐9 [17] and ICD‐9/10 codes [13], respectively, to identify metastasis had relatively similar validation estimates. However, studies that used ICD‐9 codes along with other claims‐based procedure codes from data prior to 2015 [4, 29] need to be updated and validated for ICD‐10 codes. Furthermore, algorithms that incorporate procedure codes or medication use as a part of the case definition may have limited applicability for certain research topics such as treatment patterns. For instance, if a medication is used to define the outcome, then it may not be independently assessed as an exposure. Hence, it is important to consider the components of the algorithm in the context of its use.

Some studies in our review had limited sample sizes, were specific to certain geographic regions, and a specific patient population (e.g., Medicare only). The studies also varied in terms of patient inclusion–exclusion criteria. Even though several studies did not assess the diagnostic accuracy of algorithms, we included such studies so that the researchers can have comprehensive information on all published and unpublished algorithms on relevant PC key characteristics and outcomes. In our study, one reviewer assessed the quality of included studies; however, we had two other independent reviewers validate study quality appraisal to ensure that decisions were not solely reliant on one reviewer.

## 5 Conclusion

We conclude that RWD can be used to identify key characteristics of PC and PC‐specific phenotypes, using several claims‐based algorithms and prediction models. Our findings will aid researchers who would like to identify PC patients with specific outcomes or key characteristics from EMR and/or administrative claims databases.

### 5.1 Plain Language Summary

Studies that developed algorithms to identify patients with different disease outcomes using healthcare databases need to be summarized. Many studies have reported the validity of PC outcomes identified from healthcare databases, but these studies are not systematically summarized. In this paper, we provide a comprehensive review of the healthcare studies conducted to summarize the validity of models to identify several PC outcomes, including the spread of disease. This paper provides useful information to researchers who utilize large healthcare databases to identify patients with PC with specific outcomes or disease characteristics.

## Ethics Statement

As this is a systematic review study that involves summarizing existing literature on a topic, the University of Rhode Island Institutional Review Board deemed this study nonhuman subjects research.

## Consent

The authors have nothing to report.

## Conflicts of Interest

This study was funded by Bayer Pharmaceuticals. Drs. Ami Vyas and Britny R. Brown were partially supported by this funding. Dr. Britny R. Brown also reported funding from Eli Lilly. Dr. Shweta Kamat was a graduate student at the University of Rhode Island when this study was conducted; she is now an employee of Open Health. Dr. Amit D. Raval is an employee and stockholder of Bayer Pharmaceuticals. The other authors declare no conflicts of interest.

## Supporting information


**Data S1:** pds70236‐sup‐0001‐Supinfo.pdf.

**Data S2:** pds70236‐sup‐0002‐Tables.docx.
