# Supplementary material for: Diagnostic Performance of Prostate Cancer Disease‐Specific Phenotypes Identified Using Real‐World Databases: A Systematic Review
Source: Pharmacoepidemiol Drug Saf. 2025 Oct 15;34(10):e70236. doi: 10.1002/pds.70236 (PMC12527646; doi:10.1002/pds.70236)
Supplement: Supplementary file 1 — Data S1: pds70236‐sup‐0001‐Supinfo.pdf. [file PDS-34-e70236-s002.pdf]

<u>Supplementary Material 1: Study Protocol</u>

<u>PROTOCOL TITLE</u>: Measures Used to Identify Key Disease Characteristics or Disease Progression in Prostate Cancer Using Real-World Databases: A Systematic Literature Review

1. Background and Rationale

1.1 Background

Prostate cancer is the most commonly occurring cancer in men living in the United States (1) and the second most commonly occurring cancer in men worldwide (2). It is crucial to have knowledge of the epidemiology of prostate cancer and how to monitor related outcomes for health care planning services. Administrative healthcare claims databases are widely used in the cancer therapeutic area for performing epidemiology studies (3), population-level clinical and economic outcomes studies (4-6), evaluation of health service delivery and quality (7), assess effectiveness of health policies and initiatives (8, 9), and also for health policy development (10). Administrative claims databases provide longitudinal data about healthcare utilization and costs including prescription medication use and other essential information including mortality when linked with mortality data (11). These data sources provide valuable and crucial 'real-world' information on disease surveillance and outcomes assessment in real-world patient populations (12, 13).

As administrative claims are generated for billing and reimbursement purposes, the accuracy of certain diagnoses can vary and depend on many factors such as type of patient population, provider setting, and severity of disease, to name a few. Administrative claims data lack rich and important clinical and laboratory information on disease progression, vital parameters, and performance status, which are essential for cancer research. In order to use real-world administrative data effectively, there are needs to identify proxy measures for clinical and diagnostic outcomes of cancer including disease progression and functional status. For instance, to identify a patient with prostate cancer in administrative claims, certain diagnostic codes, an algorithm/combination of criteria, diagnostic codes, or machine learning methods, must be validated according to the 'reference standard' diagnosis (14).

1.2 Rationale

In men with localized prostate cancer undergoing definitive local therapy with radical prostatectomy or radiation therapy, several men will develop disease progression measured as biochemical recurrence. If a patient initially responds to prostatectomy, as defined by an undetectable prostate specific antigen (PSA) and PSA subsequently rises to > 0.1 ng/ml or rises on 2 or more blood draws, this is defined as biochemical recurrence (15). Similarly, after radiation therapy, biochemical recurrence is defined as a rise in the PSA by > 2 ng/ml over nadir. Essentially, for measuring biochemical recurrence in real-world databases, we would require at least two measures of PSA level post-localized therapy. While administration of therapy is generally identified from the real-world databases using procedure or medication codes, PSA values are either

not available, available to a limited subset, or not available during the period to derive the operational definition. Furthermore, there are no specific International Classification of Disease Ninth or Tenth edition (ICD-9/10) codes in real-world databases to identify biochemical recurrence. However, methods to treat biochemical recurrence, such as brachytherapy, have specific diagnosis and procedure codes in real-world databases; however, brachytherapy is also used to biochemical recurrence or primary therapy for localized prostate cancer as well. Therefore, it would not be straightforward to utilize these codes to identify patients with biochemical recurrence. In this case, the operational definition of biochemical recurrence could be derived through either machine learning techniques or decision algorithm using specific criteria to derive biochemical recurrence accurately within real-world databases.

In addition to biochemical recurrence, there are other such key conditions of interest for prostate cancer real-world database research including: a) metastasis, b) progression-free survival, c) castration-resistant prostate cancer, d) castration-sensitive prostate cancer, and e) performance status/functional status.

Several validation studies have been conducted to validate the criteria of assessing metastasis (16), biochemical recurrence (17, 18), castration-resistant prostate cancer subtype (19), and functional status (20, 21) in patients with prostate cancer using the real-world administrative claims databases. However, studies on specific algorithms, criteria, and measures of prostate cancer-specific outcomes in the real-world administrative claims databases have not been systematically and comprehensively reviewed and presented using a systematic literature review approach. Such data will help researchers with identifying and utilizing appropriate and robust algorithms and criteria to assess prostate cancer-specific outcomes using administrative claims databases.

The **proposed study aims** to review the literature and systematically summarize the real-world evidence on algorithms, measures, criteria, or other such techniques of assessing prostate cancer specific outcomes including metastasis, castration resistant tumor, biochemical recurrence, progression-free survival, functional status, and spread of tumor/tumor volume.

This document describes the steps to perform a systematic literature of the real-world observational studies, starting with the method to identify and select relevant evidence from the literature by means of a systematic review.

2. Objectives

The specific research objectives are to perform a systematic literature review of real-world studies that have assessed clinical outcomes of prostate cancer using specific algorithms, measures, criteria, or other such techniques.

The followings clinical constructs will be considered in this study:

a. metastatic status
b. biochemical recurrence
c. progression-free survival
d. castration resistant/ prostate cancer
e. castration-sensitive prostate cancer
f. functional/performance status

3. Systematic Literature Search

To identify relevant real-world evidence, we will conduct a systematic literature search of the available literature as outlined in this section. We will conduct the systematic literature review in accordance with the Preferred Reporting Items for Systematic Reviews and Meta-Analysis of Diagnostic Test Accuracy Studies (PRISMA-DTA) guidelines (22).

3.1 Criteria for study inclusion/exclusion:

Based on the research objectives, study selection criteria will be defined in terms of population, index test, reference test (if available), and diagnosis of interest (PIRD criteria) to ensure that relevant studies for systematic review are identified and selected. Table 1 lists the PIRD criteria. Depending on the diagnosis of interest, the index test and the associated reference test will vary.

Table 1: PIRD criteria

Criteria	Definition
Population	Patients with prostate cancer
Index test*	An example of an algorithm to identify metastatic prostate cancer (15):<br>• Classification and regression tree (CART) models generated using the combination of variables capturing drug use, diagnosis codes, and procedures<br>• Clinical Logic-based algorithm
Reference test*	An example of reference standard to identify metastatic prostate cancer (16):<br>• Oncology Service Comprehensive Electronic records data warehouse
Diagnosis of interest	For a study to be included in the review, it should have measured at least one of the following outcomes in each study:<br>• Metastatic status<br>• Biochemical recurrence<br>• Progression-free survival<br>• Castration resistant<br>• Hormone sensitive tumor<br>• Functional status/performance status

Criteria	Definition
Study design	Real-world studies (cohort studies including retrospective and prospective cohort studies, case-control observational studies, chart reviews)
Other	No geographical restrictions;<br>Studies with sufficient details on operational definitions of the clinical constructs of interest;<br>Studies published from 2012 till present (10 years) in English language

*an example of index test and reference test are provided for a study by Nordstrom et al. (16)

3.2 Search strategy:

A comprehensive systematic search of the real-world evidence will be conducted using Medline/ PubMed and EMBASE using search strategy for the key terms for prostate cancer, cancer, and each diagnosis of interest. EMBASE captures conference abstracts and hence the grey literature in the form of conference abstracts will be identified from EMBASE. The above listed data sources will be searched from 2012 through present time point, which includes the most recent ten years of data. References of the retrieved articles will also be searched for any additional studies. The search strategy for PubMed/Medline and EMBASE are listed in Appendices 1 and 2, respectively.

3.3 Study selection:

Titles and abstracts of the studies identified by the search strategy will be reviewed by one reviewer to determine which studies are eligible for full text review based on predefined criteria. Two independent reviewers will examine full text reports of all the articles that will be deemed eligible from the prior step of titles and abstracts screening. The full text reports will be examined based on the PIRD criteria. Any disagreements between the two reviewers will be resolved through discussion and by the third reviewer.

3.4 Assessment of Study quality of included studies:

Two independent reviewers will appraise the included studies for risk of bias and applicability using the recently developed version 2 of the Quality Assessment of Diagnostic Accuracy Studies (QUADAS-2) tool (23). Use of QUADAS-2 tool is also encouraged by the current PRISMA 2020 guidance (24). Any disagreements between the two reviewers will be resolved by the third reviewer.

3.5 Data Extraction:

Data from all studies that meet the eligibility criteria will be extracted by two reviewers. Data on study characteristics including study design and inclusion-exclusion criteria, patient characteristics, outcome of interest, index test, reference test, and validity (sensitivity, specificity, predictive value) will be extracted. Any discrepancies observed with the data extraction will be resolved through discussion or by the third reviewer.

<u>3.5.1</u> Study Characteristics: We will extract the following information for study characteristics of the included studies (this list is not exhaustive):

- *Study ID*
- *Authors and year of publication,*
- *Study years and period of data included,*
- *Study location (country),*
- *Study design,*
- *Type of administrative claims data,*
- *Name of the administrative claims data,*
- *Sample size,*
- *Index test (e.g. diagnostic codes to identify metastasis),*
- *Reference test (e.g. medical records, cancer registry)*

<u>3.5.2</u> Patient population: We will extract the following information for patient characteristics of the included studies (this list is not exhaustive):

- *Study ID,*
- *Authors,*
- *Age of the patient population,*
- *Stage of disease*
- *Grade of tumor*
- *Gleason score*
- *PSA level*

<u>3.5.3</u> Diagnoses of interest: We will extract the following information for **<u>each</u>** diagnosis of interest available in the included studies (this list is not exhaustive):

- *Study ID*
- *Algorithm type (e.g. ICD-9/10 codes, prediction model)*
- *Specific Algorithm (with details allowing replication in an independent data),*
- *Sensitivity,*
- *Specificity,*
- *Positive predictive value*
- *Negative predictive value*
- *Consistency with literature clinical outcomes*

For each diagnosis of interest (e.g., metastasis, progression-free survival), an algorithm to identify each diagnosis, sensitivity, specificity, predictive values, along with the corresponding measures of uncertainty (i.e., standard errors, 95% confidence intervals) will be extracted. For each algorithm, we will also report strengths and limitations of the algorithm as described by the authors of the individual study.

If data on any characteristic or outcome is not available in an individual study, then we will contact the corresponding author of the studies to obtain missing data and information.

3.5.4 **Study Quality Assessment**: We will assess quality of included individual studies for risk of bias and applicability using the QUADAS-2) tool. We will extract the following information for each study (this list is not exhaustive):

- Risk of bias for patient selection, index test, reference standard (high, low or unclear)
- Applicability for patient selection, index test, reference standard (high, low or unclear)

4.0 Reporting of Results

After following the PIRD outlined in Table 1 and using the search strategies, we will identify relevant studies to include in the systematic review. We will utilize PRISMA flow diagram to report the included studies. After extracting the relevant data, we will develop the tables summarizing the data on study characteristics, patient population, diagnosis of interest, and study quality assessment as listed in appendices 3-6.

5.0 Limitations

Due to the nature of the systematic literature review focused on real-world studies, findings will be limited by the studies available till date of study selection, limited sample sizes, type of study design (i.e. retrospective nature), and may also be heterogeneous depending on the patient characteristics of the included studies. Given the inherent limitation of the SLR design, we plan to critically appraise and summarize study accounting for the differences.

6.0 References:

1. American Cancer Society. Cancer Facts & Figures 2023. Atlanta: American Cancer Society; 2023. Available at: https://www.cancer.org/content/dam/cancer-org/research/cancer-facts-and-statistics/annual-cancer-facts-and-figures/2023/2023-cancer-facts-and-figures.pdf Accessed May 1, 2023.
2. World Cancer Research Fund International. Worldwide cancer data. Available at: https://www.wcrf.org/cancer-trends/worldwide-cancer-data/ Accessed May 1, 2023.
3. Saltus CW, Vassilev ZP, Zong J, Calingaert B, Andrews EB, Soriano-Gabarró M, Kaye JA. Incidence of Second Primary Malignancies in Patients with Castration-Resistant Prostate Cancer: An Observational Retrospective Cohort Study in the United States. Prostate Cancer. 2019 Feb 11;2019:4387415.

4. Potosky AL, Merrill RM, Riley GF, Taplin SH, Barlow W, Fireman BH, Lubitz JD. Prostate cancer treatment and ten-year survival among group/staff HMO and fee-for-service Medicare patients. Health Serv Res. 1999 Jun;34(2):525-46.
5. Robinson TJ, Dinan MA, Li Y, Lee WR, Reed SD. Longitudinal Trends in Costs of Palliative Radiation for Metastatic Prostate Cancer. J Palliat Med. 2015 Nov;18(11):933-9.
6. Feldman AS, Meyer CP, Sanchez A, Krasnova A, Reznor G, Menon M, Kibel AS, Choueiri TK, Lipsitz SR, Sun M, Trinh QD. Morbidity and Mortality of Locally Advanced Prostate Cancer: A Population Based Analysis Comparing Radical Prostatectomy versus External Beam Radiation. J Urol. 2017 Nov;198(5):1061-1068.
7. Kern DM, Barron JJ, Wu B, Ganetsky A, Willey VJ, Quimbo RA, Fisch MJ, Singer J, Nguyen A, Mamtani R. A validation of clinical data captured from a novel Cancer Care Quality Program directly integrated with administrative claims data. Pragmat Obs Res. 2017 Aug 26;8:149-155.
8. Zhu Y, Koethe B, Ollendorf DA, Wong JB, Neumann PJ, Kim DD. Effect of the 2012 US Preventive Services Task Force Recommendations on Prostate-Specific Antigen Screening in a Medicare Advantage Population. Med Care. 2022 Dec 1;60(12):888-894.
9. Lange SM, Choudry MM, Hunt TC, Ambrose JP, Haaland BA, Lowrance WT, Hanson HA, O'Neil BB. Impact of choosing wisely on imaging in men with newly diagnosed prostate cancer. Urol Oncol. 2023 Jan;41(1):48.e19-48.e26.
10. Kim DD, Daly AT, Koethe BC, Fendrick AM, Ollendorf DA, Wong JB, Neumann PJ. Low-Value Prostate-Specific Antigen Test for Prostate Cancer Screening and Subsequent Health Care Utilization and Spending. JAMA Netw Open. 2022 Nov 1;5(11):e2243449.
11. Penberthy LT, Rivera DR, Lund JL, Bruno MA, Meyer A. An overview of real-world data sources for oncology and considerations for research. Ca Cancer J Clin 2022;72:287-300.
12. Schulman KL, Berenson K, Tina Shih YC, et al. A checklist for ascertaining study cohorts in oncology health services research using secondary data: report of the ISPOR oncology good outcomes research practices working group. Value Health 2013;16:655-669.
13. Sarrazin MS, Rosenthal GE. Finding pure and simple truths with administrative data. JAMA 2012;307:1433-1435.
14. Parlett LE, Beachler DC, Lanes S, Hoover RN, Cook MB. Validation of an Algorithm for Claims-based Incidence of Prostate Cancer. Epidemiology. 2019 May;30(3):466-471.
15. National Comprehensive Cancer Network. Prostate Cancer Guidelines V.1.2023. Available at www.nccn.org
16. Nordstrom BL, Whyte JL, Stolar M, Mercaldi C, Kallich JD. Identification of metastatic cancer in claims data. Pharmacoepidemiol Drug Saf. 2012 May;21 Suppl 2:21-8.
17. Hassett MJ, Ritzwoller DP, Taback N, Carroll N, Cronin AM, Ting GV, Schrag D, Warren JL, Hornbrook MC, Weeks JC. Validating billing/encounter codes as

indicators of lung, colorectal, breast, and prostate cancer recurrence using 2 large contemporary cohorts. Med Care. 2014 Oct;52(10):e65-73. doi: 10.1097/MLR.0b013e318277eb6f. PMID: 23222531; PMCID: PMC3600389.
18. Khan S, Vohra S, Farnan L, Elmore SNC, Toumbou K, K C M, Fontham ETH, Peters ES, Mohler JL, Bensen JT. Using health insurance claims data to assess long-term disease progression in a prostate cancer cohort. Prostate. 2022 Jul 26. doi: 10.1002/pros.24418. Epub ahead of print. PMID: 35880605.
19. Freedland SJ, Ke X, Lafeuille MH, Romdhani H, Kinkead F, Lefebvre P, Petrilla A, Pulungan Z, Kim S, D'Andrea DM, Francis P, Ryan CJ. Identification of patients with metastatic castration-sensitive or metastatic castration-resistant prostate cancer using administrative health claims and laboratory data. Curr Med Res Opin. 2021 Apr;37(4):609-622.
20. Sheffield KM, Bowman L, Smith DM, Li L, Hess LM, Montejano LB, Willson TM, Davidoff AJ. Development and validation of a claims-based approach to proxy ECOG performance status across ten tumor groups. J Comp Eff Res. 2018 Mar;7(3):193-208. doi: 10.2217/cer-2017-0040. Epub 2018 Mar 13. PMID: 29533694.
21. Davidoff AJ, Zuckerman IH, Pandya N, Hendrick F, Ke X, Hurria A, Lichtman SM, Hussain A, Weiner JP, Edelman MJ. A novel approach to improve health status measurement in observational claims-based studies of cancer treatment and outcomes. J Geriatr Oncol. 2013 Apr;4(2):157-65. doi: 10.1016/j.jgo.2012.12.005. PMID: 23795223; PMCID: PMC3685201.
22. McInnes MDF, Moher D, Thombs BD, McGrath TA, Bossuyt PM, the PRISMA-DTA Group. Preferred Reporting of Items for a Systematic Review and Meta-Analysis of Diagnostic Test Accuracy Studies: The PRISMA-DTA Statement. JAMA 2018;319(4):388-396.
23. Whiting PF, Rutjes AWS, Westwood ME, et al. QUADAS-2: a revised tool for the quality assessment of diagnostic accuracy studies. Ann Intern Med 2011;155(4):529-536.
24. Page MJ, McKenzie JE, Bossuyt PM, et al. The PRISMA 2020 statement: an updated guideline for reporting systematic reviews. BMJ 2021;372:n71.

Appendix 1: Search strategy for PubMed/Medline

#	Search terms
1	(((((((((prostate cancer) OR (prostatic cancer)) OR (prostate neoplasm)) OR (prostatic neoplasm)) OR (prostate carcinoma)) OR (prostatic carcinoma)) OR (prostate cancers)) OR (prostate)) OR (prostatic)) OR (prostate neoplasia)) OR (prostatic neoplasia)
2	(((((((cancer) OR (neoplasm)) OR (carcinoma)) OR (malignant)) OR (malignancy)) OR (neoplas*)) OR (tumor*)) OR (tumour*)
3	(((((((((((((((((((real-world evidence) OR (real-world data)) OR (real-world)) OR (RWE)) OR (RWD)) OR (real-world outcomes)) OR (health administrative)) OR (administrative data)) OR (administrative database)) OR (claim administrative)) OR (claims)) OR (electronic medical records)) OR (insurance claims)) OR (insurance administrative)) OR (registry)) OR (registries)) OR (claims-based)) OR (administrative health)) OR (administrative health claims)) OR (health insurance claims)) OR (chart reviews)) OR (chart review)
4	((((((((((((sensitivity) OR (specificity)) OR (sensitive)) OR(specific)) OR (ppv)) OR (positive predictive value)) OR (validation))OR (validat*)) OR (agreement)) OR (concordance)) OR (concordant))OR (accuracy)) OR (accurate)) OR (valid*)
5	(((metastatic[Title/Abstract]) OR (metastasis[Title/Abstract])) OR (metastases[Title/Abstract])) OR (metasta*[Title/Abstract])
6	((biochemical recurrence[Title/Abstract]) OR (recurrence[Title/Abstract])) OR (BCR[Title/Abstract])
7	((((((((castration-resistant[Title/Abstract]) OR (castration resistant[Title/Abstract])) OR (castration refractory[Title/Abstract])) OR (castration-refractory[Title/Abstract])) OR (hormone refractory[Title/Abstract])) OR (hormone-refractory[Title/Abstract])) OR (hormone resistant[Title/Abstract])) OR (hormone-resistant[Title/Abstract])) OR (hormone sensitive[Title/Abstract])) OR (hormone-sensitive[Title/Abstract])
8	((((((((progression[Title/Abstract]) AND (survival[Title/Abstract])) OR (progression-free survival[Title/Abstract])) OR (progression free survival[Title/Abstract])) OR (recurrence free survival[Title/Abstract])) OR (recurrence-free survival[Title/Abstract])) OR (disease free survival[Title/Abstract])) OR (disease-free survival[Title/Abstract])) OR (disease progression[Title/Abstract])) OR (progression[Title/Abstract])
9	(((((((('tumor volume'[Title/Abstract]) OR ('spread of tumor'[Title/Abstract])) OR ('tumor spread'[Title/Abstract])) OR ('tumor lesions'[Title/Abstract])) OR ('tumor lesion'[Title/Abstract])) OR ('tumour volume'[Title/Abstract])) OR ('tumour lesion'[Title/Abstract])) OR ('tumour lesions'[Title/Abstract])) OR

#	Search terms
	('tumor burden'[Title/Abstract])) OR ('tumour burden'[Title/Abstract])
10	((((((((((((functional status[Title/Abstract]) OR (disability status[Title/Abstract])) OR (disability[Title/Abstract])) OR (disabled[Title/Abstract])) OR (performance status[Title/Abstract])) OR (health status[Title/Abstract])) OR (well-being[Title/Abstract])) OR (physical activity[Title/Abstract])) OR (ADL[Title/Abstract])) OR (activit* of daily living[Title/Abstract])) OR (ECOG status[Title/Abstract])) OR (ECOG[Title/Abstract])) OR (PS[Title/Abstract])
11	#1 AND #3 AND #4 AND #5
12	#1 AND #3 AND #4 AND #6
13	#1 AND #3 AND #4 AND #7
14	#1 AND #3 AND #4 AND #8
15	#1 AND #3 AND #4 AND #9
16	#2 AND #3 AND #4 AND #10
17	#11 OR #12 OR #13 OR #14 OR #15 OR #16
18	Studies published during 2012-2023; English only studies

Appendix 2: Search strategy for EMBASE

#	Search terms
1	'prostate cancer'/exp OR 'prostate cancer' OR (('prostate'/exp OR prostate) AND ('cancer'/exp OR cancer)) OR 'prostatic cancer'/exp OR 'prostatic cancer' OR (prostatic AND ('cancer'/exp OR cancer)) OR 'prostate neoplasm'/exp OR 'prostate neoplasm' OR (('prostate'/exp OR prostate) AND ('neoplasm'/exp OR neoplasm)) OR 'prostatic neoplasm'/exp OR 'prostatic neoplasm' OR (prostatic AND ('neoplasm'/exp OR neoplasm)) OR 'prostate carcinoma'/exp OR 'prostate carcinoma' OR (('prostate'/exp OR prostate) AND ('carcinoma'/exp OR carcinoma)) OR 'prostatic carcinoma'/exp OR 'prostatic carcinoma' OR (prostatic AND ('carcinoma'/exp OR carcinoma)) OR 'prostate cancers' OR (('prostate'/exp OR prostate) AND ('cancers'/exp OR cancers)) OR 'prostate'/exp OR prostate OR prostatic
2	'cancer'/exp OR cancer OR 'carcinoma'/exp OR carcinoma OR malignant OR 'malignancy'/exp OR malignancy OR 'neoplasm'/exp OR neoplasm OR 'tumor'/exp OR tumor OR 'tumour'/exp OR tumour
3	'real-world evidence' OR ('real world' AND ('evidence'/exp OR evidence)) OR 'real-world data' OR ('real world' AND data) OR rwe OR rwd OR 'real-world outcomes' OR ('real world' AND ('outcomes'/exp OR outcomes)) OR 'real world' OR 'health administrative' OR (('health'/exp OR health) AND administrative) OR 'administrative data'/exp OR 'administrative data' OR

#	Search terms
	(administrative AND data) OR 'administrative database'/exp OR 'administrative database' OR (administrative AND ('database'/exp OR database)) OR claims OR 'claims administrative' OR (claims AND administrative) OR 'electronic medical records' OR (electronic AND medical AND ('records'/exp OR records)) OR 'insurance claims' OR (('insurance'/exp OR insurance) AND claims) OR 'insurance administrative' OR (('insurance'/exp OR insurance) AND administrative) OR 'registry'/exp OR registry OR 'registries'/exp OR registries OR 'claims based' OR 'administrative health' OR (administrative AND ('health'/exp OR health)) OR 'administrative health claims' OR (administrative AND ('health'/exp OR health) AND claims) OR 'health insurance claims' OR (('health'/exp OR health) AND ('insurance'/exp OR insurance) AND claims) OR 'chart reviews' OR (chart AND reviews) OR 'chart review'/exp OR 'chart review' OR (chart AND ('review'/exp OR review))
4	'sensitivity'/exp OR sensitivity OR 'specificity'/exp OR specificity OR sensitive OR specific OR ppv OR 'positive predictive value'/exp OR 'positive predictive value' OR (positive AND predictive AND ('value'/exp OR value)) OR 'validation'/exp OR validation OR 'validity'/exp OR validity OR 'accurate'/exp OR accurate OR 'accuracy'/exp OR accuracy OR 'concordance'/exp OR concordance OR concordant
5	metastasis:ab,ti OR metastases:ab,ti OR metastatic:ab,ti
6	'biochemical recurrence':ab,ti OR bcr:ab,ti OR recurrence:ab,ti
7	'castration resistant':ab,ti OR 'castration refractory':ab,ti OR 'hormone refractory':ab,ti OR 'hormone resistant':ab,ti OR 'hormone sensitive':ab,ti
8	'progression free survival':ab,ti OR 'progression-free survival':ab,ti OR 'recurrence free survival':ab,ti OR 'recurrence-free survival':ab,ti OR 'disease free survival':ab,ti OR 'disease-free survival':ab,ti OR 'disease progression':ab,ti OR progression:ab,ti
9	'spread of tumor':ab,ti OR 'spread of tumour':ab,ti OR 'tumor burden':ab,ti OR 'tumour burden':ab,ti OR 'tumor spread':ab,ti OR 'tumor lesion':ab,ti OR 'tumor volume':ab,ti OR 'tumour volume':ab,ti OR 'tumour lesion':ab,ti
10	'functional status':ab,ti OR 'disability status':ab,ti OR disabled:ab,ti OR disability:ab,ti OR 'performance status':ab,ti OR ps:ab,ti OR 'health status':ab,ti OR 'well being':ab,ti OR 'physical activity':ab,ti OR 'ecog status':ab,ti OR ecog:ab,ti
11	#1 AND #3 AND #4 AND #5
12	#1 AND #3 AND #4 AND #6
13	#1 AND #3 AND #4 AND #7
14	#1 AND #3 AND #4 AND #8
15	#1 AND #3 AND #4 AND #9

#	Search terms
16	#2 AND #3 AND #4 AND #10
17	#11 OR #12 OR #13 OR #14 OR #15 OR #16
18	Studies published during 2012-2023; English only studies; studies focusing on adult population

Abbreviations: ab: abstract; ti: title

Appendix 3: Study Characteristics

Author name, year	Study location (country) / region	Publication type (Full publication / conference proceeding)	Study design	Type of administrative claims data	Name of administrative claims data	Period of data collection	Study inclusion criteria	Study Exclusion criteria	Sample size	Source population	Type of diagnosis of interest

Appendix 4: Baseline Characteristics of Population

Author name, year	Sample size	Mean (SD) age	Race/ethnicity distribution	Cancer stage distribution	Grade of tumor	Gleason score	PSA level	Other cancer related characteristic	Other cancer related characteristic

Appendix 5: Descriptives of each diagnosis of interest

Author name, year	Study location (country) / region	Study design	Type of administrative claims data	Index test (e.g. diagnostic codes)	Algorithm type	Specific algorithm	Reference test	Sensitivity (95% CI)	Specificity (95% CI)	PPV (95% CI)	NPV (95% CI)

Appendix 6: Risk of Bias Assessment using QUADAS-2 tool

	Risk of Bias*				Applicability concerns*		
	Patient selection	Index test	Reference standard	Flow and timing	Patient selection	Index test	Reference standard
Study 1							

*will be classified as low risk, high risk, and unclear risk based on the QUADAS-2 tool
