# Supplementary material for: Diagnostic Performance of Prostate Cancer Disease‐Specific Phenotypes Identified Using Real‐World Databases: A Systematic Review
Source: Pharmacoepidemiol Drug Saf. 2025 Oct 15;34(10):e70236. doi: 10.1002/pds.70236 (PMC12527646; doi:10.1002/pds.70236)
Supplement: Supplementary file 2 — Data S2: pds70236‐sup‐0002‐Tables.docx. [file PDS-34-e70236-s001.docx]

**Supplementary Table 1: Search Strategy for PubMed**

| # | Search terms |
| --- | --- |
| 1 | ((((((((((prostate cancer) OR (prostatic cancer)) OR (prostate neoplasm)) OR (prostatic neoplasm)) OR (prostate carcinoma)) OR (prostatic carcinoma)) OR (prostate cancers)) OR (prostate)) OR (prostatic)) OR (prostate neoplasia)) OR (prostatic neoplasia) |
| 2 | (((((((cancer) OR (neoplasm)) OR (carcinoma)) OR (malignant)) OR (malignancy)) OR (neoplas*)) OR (tumor*)) OR (tumour*) |
| 3 | (((((((((((((((((((((real-world evidence) OR (real-world data)) OR (real-world)) OR (RWE)) OR (RWD)) OR (real-world outcomes)) OR (health administrative)) OR (administrative data)) OR (administrative database)) OR (claim administrative)) OR (claims)) OR (electronic medical records)) OR (insurance claims)) OR (insurance administrative)) OR (registry)) OR (registries)) OR (claims-based)) OR (administrative health)) OR (administrative health claims)) OR (health insurance claims)) OR (chart reviews)) OR (chart review) |
| 4 | (((((((((((((sensitivity) OR (specificity)) OR (sensitive)) OR(specific)) OR (ppv)) OR (positive predictive value)) OR (validation))OR (validat*)) OR (agreement)) OR (concordance)) OR (concordant))OR (accuracy)) OR (accurate)) OR (valid*) |
| 5 | (((metastatic[Title/Abstract]) OR (metastasis[Title/Abstract])) OR (metastases[Title/Abstract])) OR (metasta*[Title/Abstract]) |
| 6 | ((biochemical recurrence[Title/Abstract]) OR (recurrence[Title/Abstract])) OR (BCR[Title/Abstract]) |
| 7 | (((((((((((((castration-resistant[Title/Abstract]) OR (castration resistant[Title/Abstract])) OR (castration refractory[Title/Abstract])) OR (castration-refractory[Title/Abstract])) OR (hormone refractory[Title/Abstract])) OR (hormone-refractory[Title/Abstract])) OR (hormone resistant[Title/Abstract])) OR (hormone-resistant[Title/Abstract])) OR (hormone sensitive[Title/Abstract])) OR (hormone-sensitive[Title/Abstract])) OR (castration-naive[Title/Abstract])) OR (castration naive[Title/Abstract])) OR (castration-sensitive[Title/Abstract])) OR (castration sensitive[Title/Abstract]) |
| 8 | (((((((((progression[Title/Abstract]) AND (survival[Title/Abstract])) OR (progression-free survival[Title/Abstract])) OR (progression free survival[Title/Abstract])) OR (recurrence free survival[Title/Abstract])) OR (recurrence-free survival[Title/Abstract])) OR (disease free survival[Title/Abstract])) OR (disease-free survival[Title/Abstract])) OR (disease progression[Title/Abstract])) OR (progression[Title/Abstract]) |
| 9 | (((((((('tumor volume'[Title/Abstract]) OR ('spread of tumor'[Title/Abstract])) OR ('tumor spread'[Title/Abstract])) OR ('tumor lesions'[Title/Abstract])) OR ('tumor lesion'[Title/Abstract])) OR ('tumour volume'[Title/Abstract])) OR ('tumour lesion'[Title/Abstract])) OR ('tumour lesions'[Title/Abstract])) OR ('tumor burden'[Title/Abstract]) OR ('tumour burden'[Title/Abstract]) |
| 10 | ((((((((((((functional status[Title/Abstract]) OR (disability status[Title/Abstract])) OR (disability[Title/Abstract])) OR (disabled[Title/Abstract])) OR (performance status[Title/Abstract])) OR (health status[Title/Abstract])) OR (well-being[Title/Abstract])) OR (physical activity[Title/Abstract])) OR (ADL[Title/Abstract])) OR (activit* of daily living[Title/Abstract])) OR (ECOG status[Title/Abstract])) OR (ECOG[Title/Abstract])) OR (PS[Title/Abstract]) |
| 11 | #1 AND #3 AND #4 AND #5 |
| 12 | #1 AND #3 AND #4 AND #6 |
| 13 | #1 AND #3 AND #4 AND #7 |
| 14 | #1 AND #3 AND #4 AND #8 |
| 15 | #1 AND #3 AND #4 AND #9 |
| 16 | #2 AND #3 AND #4 AND #10 |
| 17 | #11 OR #12 OR #13 OR #14 OR #15 OR #16 |
| 18 | Studies published during 2012-2023; English only studies |

**Supplementary Table 2: Additional Characteristics of the Included Studies**

| **Study ID** | **Phenotypes** | **Age of the Patients (Years)** | **Study Inclusion Criteria** | **Study Exclusion Criteria** |
| --- | --- | --- | --- | --- |
| Abdul et al., 2019 | BCR | NR | Enrolled in cancer registry and diagnosed with PC, and received RT up to 74Gy | - |
| Alba et al., 2021 | M1 | Mean: 71.2 (for entire population); Non metastatic: 71.3 (SD: 9.4); Metastatic: 69.7 (SD: 9.6) | With a diagnosis of PC via at least: (1) ICD-9/10 procedure codes and CPT codes for prostate biopsy, (2) ICD-9/10 diagnosis codes of PC, and (3) patients with PC as their primary site of tumor | - |
| Arnold et al., 2019 | nmCRPC | NR | Diagnosed with PC | - |
| Bai et al., 2021 | BM | Median: 72-74 | Histologically newly diagnosed with PC for the first time | History of hormone therapy at the time of staging imaging or a history of 5-alpha reductase inhibitor use |
| Danciu et al., 2022 | BCR | NR | With localized PC (N0 and M0) at diagnosis, clinical Gleason score of 6-10, and with baseline PSA between 1-100 ng/ml | Without a biopsy confirmed diagnosis, who had another primary cancer either before or after the PC diagnosis |
| Davidoff et al., 2013 | PS/HS/DS | All >=65 | Adults enrolled in Medicare including non-elderly adults with disabilities (also included non-cancer patients) | With any Medicare Advantage enrollment or who resided in facilities other than assisted living/nursing homes |
| Dolan et al., 2012 | M1 | NR | Admitted to the hospital who had an ICD-9 diagnosis code of PC | Without available medical charts |
| Dong et al., 2022 | BM | <75 years (89.7%), >=75 years (10.3%) | Diagnosed with PC identified using ICD-10 diagnosis and ICD-0-3 histology codes | Diagnosed only by autopsy/death certificate, without histological confirmation, with more than one primary tumor, with incomplete follow-up and baseline demographic data, with unknown/ incomplete clinicopathological data |
| Du et al., 2020 | nmHSPC, mHSPC, nmCRPC,  mCRPC | NR | With PC | - |
| Ehrenstein et al., 2015 | BM,  DM,  M1 | Mean: 70.5 | Diagnosed with PC using ICD-10 diagnosis code | With diagnostic codes for distant metastasis on or after PC diagnosis date, or with other cancer |
| Freedland et al., 2021 | mCSPC, mCRPC | NR | Adult males with ≥2 claims with PC diagnosis via ICD-9/10 diagnosis codes, ≥1 claim for metastatic cancer diagnosis on or after the first PC diagnosis, no claims for metastatic disease prior to the first PC diagnosis, no claims for other cancer types prior to the first metastatic disease observed on or after the first observed PC diagnosis, ≥12 months of continuous enrollment prior to claim indicating CR/CS, ≥30 days of follow-up post claim indicating CR/CS | - |
| Hassett et al., 2014 | BCR | >=65 (40%) | Age 21 diagnosed with a first nonmetastatic PC, had definitive local therapy within 12 months of diagnosis, were documented free of disease after completing initial therapy, no death from cancer diagnosis without documented recurrence | Locally advanced or primarily progressive disease, did not complete definitive local-regional therapy, with missing medical record abstraction data, with unknown recurrence status, who had documentation of recurrent disease before they had definitive local therapy |
| Hu et al., 2014 | BCR | Median: 63 (IQR: 59-66) | With PC who underwent laparoscopic RP | Lost to follow-up, used neo-adjuvant hormonal therapy, or had incomplete datasets |
| Jeong et al., 2012 | LNM | Mean: 65.3 | With clinical stage T1c-T2a PC, were treated solely with RP | With disqualified medical records/missing data |
| Leapman et al., 2023 | BCR,  M1 | NR | With Decipher prostate genomic classifier who were successfully linked to Clarivate real world database | - |
| Liu et al., 2021 | BM | <60 years (25.4%), >=60 years (74.6%) | Diagnosed with PC | PC was not the first tumor, missing data on race, grade, PSA, Gleason score, T stage, N stage, metastatic status, and marital status |
| Malone et al., 2022 | nmCRPC | NR | Males diagnosed with PC, age >=66 years at diagnosis, with history of ADT prescription from 30 days prior to PC diagnosis onwards to end of study period, continuous ADT use with no more than a gap of 30 days between two prescriptions, and with a diagnosis of CR | Concomitant RT with ADT, PSA value >20 ng/ml within 90 days prior to first continuous ADT, not eligible for insurance coverage during two years prior to diagnosis |
| Nordstrom et al., 2012 | M1 | Mean for metastatic cases: 71.4 (SD=9.4); Mean for non-metastatic cases: 69.8 (SD=8.7) | Diagnosed with PC identified using ICD-9 diagnosis code, with at least one valid cancer stage recorded, and with medical claims data available | With multiple primary tumor types, with claims ended or cancer stage changed <= 60 days after the date of the most advanced stage recorded, with no cancer diagnosis claims from a specialist within 2 days before or after the date of the most advanced stage recorded, had <2 visits to the specialist who issued the qualifying claims of cancer diagnosis, if the specialist did not continuously report claims for at least 6 months before and 2 months after the patient's index date, with no pharmacy claims after at least one order for oral chemotherapy |
| Onukwugha et al., 2014 | BM | 66-74 years (32.3%), >74 years (67.7%) | Age >=66 and diagnosed with incident PC, with stage IV disease, with continuous enrollment in Medicare parts A/B during the 12 months prior to and including the month of cancer diagnosis | With HMO enrollment during the 12 months prior to and including the month of PC diagnosis, with history of other cancers within 5 years prior to PC diagnosis |
| Preisser et al., 2020 | M1 | Median: 65 (IQR: 59-71) | Age <=90 years with histologically confirmed PC (as first or only cancer) and with available data on PSA, clinical T-stage, and GGG | With clinical tumor stages T0 or Tx, and unknown metastatic stage |
| Sabbagh et al., 2023 | LNM | Median: 64 | Adults with PC treated with RP and pelvic lymph node dissection | Received neoadjuvant ADT, with missing information on outcome, biopsy, core positives, PSA, or lymph nodes removed, with cT4, and with PSA <0.1 ng/ml or >50 ng/ml |
| Sathiakumar et al., 2017 | BM | All >=65 | With diagnosis of PC via an ICD-9 diagnosis code, with Medicare parts A/B coverage as of the first cancer claim, age >=65 as of the first cancer claim | Enrolled in HMO as of the first cancer claim |
| Sheffield et al., 2018 | PS | Mean: 64 (SD: 12.7) | With an ICD-9 diagnosis code for cancer and at least one additional encounter for the same cancer type, with a recorded PS measure on or within 30 days of the earliest encounter with a relevant cancer diagnosis, had linkage to the MarketScan data, continuous enrollment with medical and pharmacy benefits within 12 months prior to PS measurement date | - |
| Shui et al., 2022 | M1 | NR | Adult males diagnosed with PC and who had a chart review data | - |
| Thomsen et al., 2020 | M1 | <=60 (16%), 61-70 (42%) 71-80 (30%), >80 (12%) | Diagnosed with incident PC | - |
| Thurin et al., 2021 | mCRPC | NR | Men age >=40 and with no gap >1 year in their data, covered by the general health insurance scheme, with PC identified by the presence of at least: 1) registration for PC as a long-term disease prior to 2014, 2) reimbursements for ADT for at least 2 months, 3) at least one reimbursement of estamustine or MCRPC-specific treatments, and 4) a hospital stay for PC including notably chemotherapy or RT, and a PC-specific procedure or treatment in the 5-year history period (RP, orchiectomy, HIFU, brachytherapy, cryotherapy) | Chemically castrated for a reason other than PC, especially paraphilia, with exclusive dispensing of leuproreline, triptoreline or cyproterone in the 5-year history period with less than three PSA tests, without long term disease registration or hospital stays for PC and with a long term disease registration for persistent delusional disorders, specific personality disorders, unspecified mental retardation, or gender identity disorders |
| Vincini et al., 2023 | BCR | NR | With PC who had undergone multiparametric MRI of the prostate and prostatectomy | - |
| Xiang et al., 2021 | BCR,  DM | Median: 66 (IQR: 60-72) | Treated for high/very risk PC (PSA >20 ng/ml, Gleason score 8-10, or clinical stage T3 to T4), who did not have nodal or metastatic disease on conventional imaging and curative-intent treatment with RP, EBRT, or EBRT+brachytherapy, with or without ADT, and with known follow-up and vital status | - |
| Yang et al., 2022 | M1 | <60 years (14.6%),  60-69 (37.7%),  70-79 (30.9%), >=80 (16.8%) | With PC and radiology (bone scan, CT, or MRI) reports | - |

Abbreviations: PC: Prostate Cancer; NR: Not Reported; BM: Bone Metastasis; HS/DS: Health Status/Disability Status; NMCRPC: Non-Metastatic Castration-Resistant Prostate Cancer; MCRPC: Metastatic Castration-Resistant Prostate Cancer; NMHSPC: Non-Metastatic Hormone-Sensitive Prostate Cancer; MHSPC: Metastatic Hormone-Sensitive Prostate Cancer; MCSPC: Metastatic Castration-Sensitive Prostate Cancer; BCR: Biochemical Recurrence; LNM: Lymph Node Metastasis; PS: Performance Status; RT: Radiation Therapy; SD: Standard Deviation; IQR: Interquartile Range; ICD: International Classification of Diseases; CPT: Current Procedural Terminology; PSA: Prostate Specific Antigen; ng: Nanogram; CR: Castration-Resistance; CS: Castration-Sensitivity; RP: Radical Prostatectomy; ADT: Androgen Deprivation Therapy; HMO: Health Maintenance Organization; GGG: Gleason Grade Group; HIFU; High-Intensity Focused Ultrasound; MRI: Magnetic Resonance Imaging; EBRT: External Beam Radiation Therapy; CT: Computed Tomography

**Supplementary Table 3: Detailed Description of Diagnostic Performance of Biochemical Recurrence Phenotype**

| **Study ID** | **Algorithm Type /Index Test** | **Specifics of an Algorithm / Index Test** | **Reference Standard** | **Sensitivity (95% CI)** | **Specificity (95% CI)** | **PPV (95% CI)** | **AUC (95% CI)** | **Proportion (95% CI) of Patients with an Outcome of Interest** |
| --- | --- | --- | --- | --- | --- | --- | --- | --- |
| ***Algorithms Based on Claims-Based Codes*** | | | | | | | | |
| 1. ICD codes OR Medication codes | | | | | | | | |
| Hassett et al., 2014 | ICD codes / HCPCS codes/ CPT codes/NDC codes/DRG codes | 1. ICD-9 codes of 197-198.82 for secondary malignant neoplasm for the site other than the original cancer, **OR** 2. Chemotherapy use (identified from procedure codes for the chemotherapy administration and medication codes for specific chemotherapy drugs, ICD-9 codes, DRG codes, CPT codes, HCPCS codes, and NDCs) | Chart review | 6.0% (1.0%-27.0%)^§^; 19.0% (12.0%-30.0%)^$^ | 97.0% (96.0%-99.0%)^§^; 83.0% (80.0%-86.0%)^$^ | 3.0%^§^; 11.0%^$^ | NR | NR |
| 1. ICD codes only | | | | | | | | |
| Hassett et al., 2014 | ICD codes | ICD-9 codes of 197-198.82 for secondary malignant neoplasm for the site other than the original cancer | Chart review | 0.0% (0.0%-18.0%)^§^; 10.0% (5.0%-19.0%)^$^ | 99.0% (98.0%-100.0%)^§^; 98.0% (96.0%-98.0%)^$^ | 0.0%^§^; 31.0%^$^ | NR | NR |
| Leapman et al., 2023^¥^ | ICD codes | ICD-10 code of R97.21 | Clinical information obtained during Decipher testing | NR | NR | NR | NR | NR |
| 1. Medication use only | | | | | | | | |
| Hassett et al., 2014 | ICD codes / HCPCS codes/ CPT codes/NDC codes/DRG codes | Chemotherapy use (identified from procedure codes for the chemotherapy administration and medication codes for specific chemotherapy drugs, ICD-9 codes, DRG codes, CPT codes, HCPCS codes, and NDCs) | Chart review | 6.0% (1.0%-27.0%)^§^; 3.0% (1.0%-9.0%)^$^ | 98.0% (97.0%-98.0%)^§^; 98.0% (97.0%-99.0%)^$^ | 4.0%^§^; 13.0%^$^ | NR | NR |
| ***Algorithms Based on Prediction Models*** | | | | | | | | |
| Hu et al., 2014 | GS | Prediction based on GS | Clinical information about BCR obtained from PSA values | NR | 8.9% (7.4%-10.6%) ^ψ^ | NR | 71.5% (68.0%-75.0%) | NR |
| Hu et al., 2014 | PSA | Prediction based on PSA value | Clinical information about BCR obtained from PSA values | NR | 18.6% (16.5%-20.8%) ^β^; 8.5% (7.0%-10.1%) ^ψ^ | NR | 61.9% (58.2%-65.7%) | NR |
| Hu et al., 2014 | ANN prediction model | Model included age, PSA, % free PSA, prostate weight, DRE status, pathological stage, margin status, GS | Clinical information about BCR obtained from PSA values | NR | 35.1% (32.6%-37.8%) ^β^; 20.0% (17.9%-22.3%) ^ψ^ | NR | 75.4% (72.1%-78.6%) | NR |
| Hu et al., 2014 | LR prediction model | Model included age, PSA, % free PSA, prostate weight, DRE status, pathological stage, margin status, GS | Clinical information about BCR obtained from PSA values | NR | 36.5% (33.9%-39.2%) ^β^; 18.8% (16.8%-21.1%) ^ψ^ | NR | 75.5% (72.3%-78.7%) | NR |
| Danciu et al., 2022 | ML algorithm | Algorithm included age at diagnosis, race, ethnicity, clinical GS, AJCC stage group, SEER summary stage, computed stage value, PSA values (aggregated into minimum, maximum, average, density, SD values across the 5-year period prior to diagnosis), PSA values before diagnosis adjudicated over the last year, the penultimate PSA over the last 5 years, and their rate of change | NR | NR | NR | NR | 75.7% (75.6%-75.7%) | < 5 years (5.9%);  5-10 years (2.6%); >10 years (0.6%) |
| Vincini et al., 2023 | GBDT model | Model included clinical features (age, comorbidities, risk class, PSA, clinical T-stage, clinical N-stage, pre-operative GS, pre-operative ISUP) | NR | NR | NR | NR | 59.0% | NR |
| Abdul et al., 2019 | NP model | Model included baseline PSA level, T-stage, N-stage, GS (primary, secondary, and total) | NR | NR | NR | NR | NR | 98.5% (96.5%- 100.0%)^@^; 91.7% (87.0%- 96.4%)^$^ |
| Xiang et al., 2021 | NP model | Model included initial PSA level, biopsy GGG, % positive cores, and clinical T-stage | Chart review | NR | NR | NR | 63.0% (61.0%-65.0%) | NR |

^@^ At two years follow-up

^$^ At five years follow-up

^§^ At 14 months follow-up

^β^ At 90% sensitivity

^ψ^ At 95% sensitivity

^¥^ Concordance value of 96.3%

Abbreviations: NP: Nomogram Prediction; PPV: Positive Predictive Value; NPV: Negative Predictive Value; AUC: Area Under the Curve; CI: Confidence Intervals; NR: Not Reported; PC: Prostate Cancer; ICD: International Classification of Diseases; PSA: Prostate Specific Antigen; GGG: Gleason Grade Group; GS: Gleason Score; DRE: Digital Rectal Exam; CPT: Current Procedural Terminology; HCPCS: Healthcare Common Procedure Coding System; NDC: National Drug Codes; DRG: Diagnosis-Related Group; ISUP: International Society of Urological Pathology; ML: Machine Learning; ANN: Artificial Neural Networks; GBDT: Gradient Boosted Decision Tree; LR: Logistic Regression; AJCC: American Joint Committee on Cancer; SD: Standard Deviation; BCR: Biochemical Recurrence

**Supplementary Table 4: Detailed Description of Diagnostic Performance of Metastases Phenotypes**

| **Study ID** | **Algorithm Type /Index Test** | **Specifics of an Algorithm / Index Test** | **Reference Standard** | **Accuracy (95% CI)** | **Sensitivity (95% CI)** | **Specificity (95% CI)** | **PPV (95% CI)** | **NPV (95% CI)** | **AUC (95% CI)** |
| --- | --- | --- | --- | --- | --- | --- | --- | --- | --- |
| **Any Metastasis** | | | | | | | | | |
| ***Algorithms Based on Prediction Models*** | | | | | | | | | |
| Preisser et al., 2020 | LR model | Model included GGG, PSA, and clinical tumor stage | SEER-based measure of metastasis | NR | NR | NR | NR | NR | 94.3% (94.2%-94.3%) |
| Thomsen et al., 2020 | Prediction model | Model included age at diagnosis, year of diagnosis, mode of detection, clinical TNM, serum PSA level, clinical T-stage, GGG, primary treatment, CCI, educational level, marital status | Registry data | NR | NR | NR | NR | NR | 88.0% |
| ***Algorithms Based on Combination of Claims-Based Codes and Laboratory Values*** | | | | | | | | | |
| Ehrenstein et al., 2015 | ICD codes/Procedure codes/therapy codes/lab values | ICD-10 code of C77-C79 + High PSA level (>50 ng/ml) in the absence of bone scintigraphy or antiresorptive therapy | Chart review | NR | NR | NR | 3.0% (0.07%-14.0%) ^@^; 16.0% (6.0%-32.0%)^$^ | NR | NR |
| Ehrenstein et al., 2015 | ICD codes/Procedure codes/therapy codes/lab values | ICD-10 code of C77-C79 + High PSA level (>50 ng/ml) combined with bone scintigraphy | Chart review | NR | NR | NR | 12.0% (4.0%-29.0%)^@^; 28.0% (14.0%-47.0%)^$^ | NR | NR |
| Ehrenstein et al., 2015 | ICD codes/Procedure codes/therapy codes/lab values | ICD-10 code of C77-C79 + Low PSA level (≤50 ng/ml) combined with either bone scintigraphy or antiresorptive therapy | Chart review | NR | NR | NR | 10.0% (3.0%-24.0%)^@^; 15.0% (6.0%-30.0%)^$^ | NR | NR |
| ***Algorithms Based on Claims-Based Codes*** | | | | | | | | | |
| 1. ICD codes only | | | | | | | | | |
| Leapman et al., 2023 | ICD codes/CPT codes/pharmacy codes/SNOMED clinical terms | Diagnosis codes for metastasis | Clinical information from Decipher testing | 94.9% | NR | NR | NR | NR | NR |
| Alba et al., 2021 | ICD codes | Secondary malignant neoplasm on or after first PC diagnosis identified through ICD-9 codes of 198.1, 198.5, 196.5, 196.6 or ICD-10 codes of C79.11, C79.51, C79.52, C77.4, C77.5 | Chart review | NR | 80.9% | 95.3% | 87.5% | 87.5% | NR |
| Dolan et al., 2012^ɸ^ | ICD codes | ICD-9 codes of 198.5 (bone and bone marrow metastasis) or 197.0 (lung metastasis) or 197.7 (liver metastasis) or 198.3 (brain and spinal cord metastasis) | Chart review | NR | 95.0% (80.0%-96.0%) | 100.0% (98.0%-100.0%) | 100.0% (94.0%-100.0%) | 98.7% (96.0%-99.0%) | NR |
| Dolan et al., 2012^β^ | ICD codes | ICD-9 code of 198.5 (bone and bone marrow metastasis) | Chart review | NR | 90.0% (80.0%-96.0%) | 100.0% (98.0%-100.0%) | 100.0% (94.0%-100.0%) | 97.5% (95.0%-99.0%) | NR |
| Yang et al., 2022 | ICD codes | ICD-9 (196, 196.1, 196.2, 196.3, 196.5, 196.6, 196.8, 196.9, 197, 197.1, 197.2, 197.3, 197.4, 197.5, 197.6, 197.7, 197.8, 198, 198.1, 198.2, 198.3, 198.4, 198.5, 198.6, 198.7, 198.81, 198.82, 198.89, 199, 199.1, 199.2) and ICD-10 (C77.0, C77.1, C77.2, C77.3, C77.4, C77.5, C77.8, C77.9, C78.0, C78.00, C78.01, C78.02, C78.1, C78.2, C78.39, C78.4, C78.5, C78.6, C78.7, C78.89, C79.00, C79.01, C79.02, C79.10, C79.11, C79.19, C79.2, C79.31, C79.32, C79.40, C79.49, C79.51, C79.52, C79.60, C79.61, C79.62, C79.70, C79.71, C79.72, C79.81, C79.82, C79.89, C79.9, C7B.00, C7B.01, C7B.02, C7B.03, C7B.04, C7B.09, C7B.1, C7B.8) | Hand abstracted data from radiology reports | NR | 73.0% | 86.0% | 78.0% | 82.0% | NR |
| Shui et al., 2022 | ICD codes | ICD-9/10 codes for secondary malignant neoplasms | Chart review | NR | 72.9% (65.7%-79.3%) | 91.7% (89.1%-93.9%) | 49.5%-79.1% for mPC prevalence of 10-30% | 88.8% to 96.8% for mPC prevalence of 10-30% | NR |
| 1. ICD codes OR Prescription use | | | | | | | | | |
| Alba et al., 2021 | Prescription codes **OR** ICD codes | Prescription of abiraterone or enzalutamide **OR** Secondary malignant neoplasm on or after first PC diagnosis identified through ICD-9 codes of 198.1, 198.5, 196.5, 196.6 or ICD-10 codes of C79.11, C79.51, C79.52, C77.4, C77.5 | Chart review | NR | 89.7% | 92.1% | 92.6% | 92.6% | NR |
| Nordstrom et al., 2012 | ICD codes/CPT codes/HCPCS codes/NDC codes | Evidence of the following from the date of the most advanced stage through 60 days: 1) ICD-9 diagnosis codes of 197.xx-198.xx for secondary neoplasm **OR** ICD-9 diagnosis code of 199.xx for unspecified tumors that could be either primary or secondary, **OR**  2) Metastatic agent including bicalutamide, flutamide, megestrol acetate, docetaxel, leuprolide acetate, triptorelin pamoate, zoledronic acid, pegfilgrastim, gemcitabine HCl, filgrastim, pamidronate, identified using NDC, HCPCS codes, CPT codes | EHR recorded cancer stage | NR | 81.0% | 75.0% | 86.0% | 67.0% | 82.0% |
| Shui et al., 2022 | ICD codes/ treatment codes (ICD/CPT/HCPCS) | ICD-9/10 codes for secondary malignant neoplasm **AND/OR** common metastatic prostate cancer treatments | Chart review | NR | 80.2% (73.6%-85.8%) | 88.8% (85.9%-91.3%) | 44.3%-75.4% for mPC prevalence of 10-30% | 91.3% to 97.6% for mPC prevalence of 10-30% | NR |
| 1. Prescription use only | | | | | | | | | |
| Alba et al., 2021 | Prescription codes | Prescription of abiraterone or enzalutamide | Chart review | NR | 59.6% | 99.5% | 77.6% | 77.6% | NR |
|  |  |  |  |  |  |  |  |  |  |
| **Bone Metastasis** | | | | | | | | | |
| ***Algorithms Based on Prediction Models*** | | | | | | | | | |
| Bai et al., 2021 | NP model | Model included age, clinical tumor stage, total PSA, GS, prostate volume, red cell distribution width, serum alkaline phosphatase, neutrophil/lymphocyte percentage | Positive bone scan **OR** CT **and/or** MRI if bone scan was ambiguous | NR | NR | NR | NR | NR | 95.8% (93.0%-98.0%) |
| Dong et al., 2022 | NP model | Model including age, race, marital status, grade, PSA, ISUP, T stage, N stage, brain metastasis, liver metastasis, lung metastasis | SEER based measure of BM | NR | NR | NR | NR | NR | 95.0%^§^; 94.8%^¶^ |
| Liu et al., 2021 | ML model (XGB) | Model included age at diagnosis, race, grade, PSA value, GS, T-stage, N-stage, marital status | SEER based measure of incident BM | 88.1%^£^; 88.4%^¥^ | 90.5%^£^; 90.6%^¥^ | 88.0%^£^; 87.9%^¥^ | NR | NR | 95.5% ^£^; 96.2%^¥^ |
| Liu et al., 2021 | ML model (DT) | Model included age at diagnosis, race, grade, PSA value, GS, T stage, N stage, marital status | SEER based measure of incident BM | 83.3% ^£^; 87.7%^¥^ | 88.3% ^£^; 84.6%^¥^ | 83.1% ^£^; 88.4%^¥^ | NR | NR | 93.8% ^£^; 94.4%^¥^ |
| Liu et al., 2021 | ML model (RF) | Model included age at diagnosis, race, grade, PSA value, GS, T stage, N stage, marital status | SEER-based measure of incident BM | 87.9%^£^ 87.4%^¥^ | 90.2%^£^; 88.0%^¥^ | 87.9%^£^ 87.3%^¥^ | NR | NR | 95.0%^£^; 94.9%^¥^ |
| Liu et al., 2021 | ML model (MLP) | Model included age at diagnosis, race, grade, PSA value, GS, T stage, N stage, marital status | SEER-based measure of incident BM | 87.6%^£^; 87.4%^¥^ | 89.8%^£^; 90.6%^¥^ | 87.5%^£^; 86.7%^¥^ | NR | NR | 94.7%^£^; 95.0%^¥^ |
| Liu et al., 2021 | ML model (LR) | Model included age at diagnosis, race, grade, PSA value, GS, T stage, N stage, marital status | SEER-based measure of incident BM | 84.9%^£^; 84.9%^¥^ | 86.7%^£^; 86.7%^¥^ | 84.8%^£^; 84.8%^¥^ | NR | NR | 90.3%^£^; 90.5%^¥^ |
| Liu et al., 2021 | ML model (NBC) | Model included age at diagnosis, race, grade, PSA value, GS, T stage, N stage, marital status | SEER-based measure of incident BM | 88.0%^£^; 86.9%^¥^ | 88.5%^£^; 91.4%^¥^ | 87.9%^£^; 85.9%^¥^ | NR | NR | 94.1%^£^; 93.4%^¥^ |
| ***Algorithms Based on Claims-Based Codes and Laboratory Values*** | | | | | | | | | |
| Ehrenstein et al., 2015 | ICD codes/Procedure codes/lab values | ICD-10 code of C77-C79 + High PSA level (>50 ng/ml) combined with bone scintigraphy | Chart review | NR | NR | NR | 3.0% (0.08%-16.0%)^@^; 9.0% (2.0%-25.0%)^$^ | NR | NR |
| Ehrenstein et al., 2015 | ICD codes/Procedure codes/therapy codes/lab values | ICD-10 code of C77-C79 + Low PSA level (≤50 ng/ml) combined with either bone scintigraphy or antiresorptive therapy | Chart review | NR | NR | NR | 5.0% (0.6%-17.0%)^@^; 5.0% (1.0%-17.0%)^$^ | NR | NR |
| Ehrenstein et al., 2015 | ICD-codes/Procedure codes/therapy codes/lab values | ICD-10 code of C77-C79 + High PSA level (>50 ng/ml) in absence of bone scintigraphy or antiresorptive therapy | Chart review | NR | NR | NR | 3.0% (0.07%-14.0%)^@^; 11.0% (3.0%-25.0%)^$^ | NR | NR |
| ***Algorithms Based on Claims-Based Codes*** | | | | | | | | | |
| Onukwugha et al., 2014 | ICD codes | At least one claim with an ICD-9 diagnosis code of 198.5 for secondary malignant neoplasm of bone and bone marrow in any diagnosis field | SEER-based measure of incident BM | NR | 59.8% (57.4%-62.1%) | 53.8% (50.7%-56.9%) | 68.4% (66.0%-70.8%) | NR | NR |
| Onukwugha et al., 2014 | ICD codes/CPT codes/ HCPCS codes | 1) At least one inpatient claim with an ICD-9 diagnosis code of 198.5 for secondary malignant neoplasm of bone and bone marrow as the primary or secondary discharge diagnosis, **OR**  2) At least one outpatient claim with an ICD-9 diagnosis code of 198.5 + a code for procedure used to diagnose or treat bone metastasis such as bone scan, bone biopsy, and/or use of intravenous bisphosphonate, **OR** 3) At least one outpatient claim with an ICD-9 diagnosis code of 198.5 | SEER based measure of incident BM | NR | 55.5% (53.1%-57.9%) | 58.4% (55.3%-61.4%) | 69.0% (66.5%-71.5%) | NR | NR |
| Onukwugha et al., 2014 | ICD codes | 1) At least one inpatient claim with an ICD-9 diagnosis code of 198.5 for secondary malignant neoplasm of bone and bone marrow in any diagnosis field, **OR**  2) At least two outpatient claims with an ICD-9 diagnosis code of 198.5 within a 90-day window | SEER based measure of incident BM | NR | 48.0% (45.6%-50.4%) | 62.0% (59.0%-65.0%) | 67.9% (65.1%-70.5%) | NR | NR |
| Sathiakumar et al., 2017^ψ^ | ICD-9 codes/CPT codes/HCPCS code | ICD-9 diagnosis code of 198.5 for BM **AND** at least one of the following: 1) At least one inpatient claim for primary diagnosis of BM (ICD-9: 198.5), 2) At least one inpatient claim with a primary or secondary diagnosis of BM (ICD-9: 198.5), 3) At least one physician or outpatient hospital claims with BM codes + CPT codes for specified treatment (radiation therapy for BM, bone surgery, fracture other than spine, spine fracture, changes in chemotherapy) in the same claim,  4) Physician or outpatient hospital claim with BM codes + CPT codes for specified diagnostic procedure (spinal cord compression, bone biopsy, bone scan, X-rays of pelvis/femur, MRI of back, PET scan, bone pain, high alkaline phosphatase plus hypercalcemia) in the same claim,  5) Physician or outpatient hospital claim with BM codes + evaluation and management CPT codes in the same claim | Chart review | NR | 91.7% (78.2%-97.1%) | 98.5% (95.7%-99.5%) | 91.7% (78.2%-97.1%) | 98.5% (95.7%-99.5%) | NR |
| Sathiakumar et al., 2017^Ϣ^ | ICD-9 codes/CPT codes/HCPCS code | ICD-9 diagnosis code of 198.5 for BM **AND** at least one of the following: 1) At least one inpatient claim for primary diagnosis of BM (ICD-9: 198.5), 2) At least one inpatient claim with a primary or secondary diagnosis of BM (ICD-9: 198.5), 3) At least one physician or outpatient hospital claims with BM codes + CPT codes for specified treatment (radiation therapy for BM, bone surgery, fracture other than spine, spine fracture, changes in chemotherapy) in the same claim,  4) Physician or outpatient hospital claim with BM codes + CPT codes for specified diagnostic procedure (spinal cord compression, bone biopsy, bone scan, X-rays of pelvis/femur, MRI of back, PET scan, bone pain, high alkaline phosphatase plus hypercalcemia) in the same claim,  5) Physician or outpatient hospital claim with BM codes + evaluation and management CPT codes in the same claim | Chart review | NR | 73.3% (59.0%-84.0%)** | 99.6% (98.9%-99.9%)** | 91.7% (78.2%-97.1%)** | 98.5% (97.4%-99.1%)** | NR |
|  |  |  |  |  |  |  |  |  |  |
| **Lymph Node Metastasis** | | | | | | | | | |
| ***Algorithms Based on Prediction Models*** | | | | | | | | | |
| Jeong et al., 2012 | NP model | Model included prebiopsy serum PSA, clinical stage, biopsy GS, percent positive cores | Pathological assessment of prostatectomy specimen | NR | NR | NR | NR | NR | 83.8%^£^; 96.5%^¥^ |
| Sabbagh et al., 2023 | ML model (SLR) | Model included age at diagnosis, PSA at diagnosis or before treatment, clinical T-stage, percentage positive cores, primary and secondary biopsy GS | NR | NR | NR | NR | NR | NR | 81.0% (80.0%-82.0%)^£^; 81.0%^¥^ |
| Sabbagh et al., 2023 | ML model (LRE model) | Model included age at diagnosis, PSA at diagnosis or before treatment, clinical T-stage, percentage positive cores, primary and secondary biopsy GS | NR | NR | NR | NR | NR | NR | 81.0% (80.0%-82.0%)^£^; 81.0%^¥^ |
| Sabbagh et al., 2023 | ML model (XGB) | Model included age at diagnosis, PSA at diagnosis or before treatment, clinical T-stage, percentage positive cores, primary and secondary biopsy GS | NR | NR | NR | NR | NR | NR | 82.0% (81.0%-83.0%) ^£^; 82.0%^¥^ |
|  |  |  |  |  |  |  |  |  |  |
| **Distant Metastasis** | | | | | | | | | |
| ***Algorithms Based on Combination of Claims-Based Codes and Laboratory Values*** | | | | | | | | | |
| Ehrenstein et al., 2015 | ICD codes/Procedure codes/therapy codes/lab values | Distant non-bone metastasis: ICD-10 code of C77-C79 + High PSA level (>50 ng/ml) in the absence of bone scintigraphy or antiresorptive therapy | Chart review | NR | NR | NR | 5.0% (1.0%-18.0%)^$^ | NR | NR |
| Ehrenstein et al., 2015 | ICD codes/Procedure codes/therapy codes/lab values | Distant non-bone metastasis: ICD-10 code of C77-C79 + High PSA level (>50 ng/ml) combined with bone scintigraphy | Chart review | NR | NR | NR | 9.0% (2.0%-25.0%)^@^; 19.0% (7.0%-36.0%)^$^ | NR | NR |
| Ehrenstein et al., 2015 | ICD codes/Procedure codes/therapy codes/lab values | Distant non-bone metastasis: ICD-10 code of C77-C79 + Low PSA level (<=50 ng/ml) combined with either bone scintigraphy or antiresorptive therapy | Chart review | NR | NR | NR | 5.0 (0.6%-17.0%)^@^; 10.0% (3.0%-24.0%)^$^ | NR | NR |
| ***Algorithms Based on Prediction Models*** | | | | | | | | | |
| Xiang et al., 2021 | NP model | Model included initial PSA level, biopsy GGG, percentage positive cores, and clinical T-stage | Medical records | NR | NR | NR | NR | NR | 69.0% (66.0%-71.0%) |

^ɸ^ Kappa statistic of 96.8% (95% CI: 93.0%-100.0%)

^β^ Kappa statistic of 93.5% (95% CI: 88.0%-99.0%)

^ψ^ Kappa statistic of 90.0% (95% CI: 77.0%-100.0%)

^Ϣ^ Kappa statistic of 81.0% (95% CI: 74.0%-87.0%)

^@^ 30 days before or after the index date

^$^ 180 days before or after the index date

^§^ Training dataset

^¶^ Validation dataset

^£^ Internal validation

^¥^ External validation

** Weighted findings

Abbreviations: NLP: Natural Language Processing; PPV: Positive Predictive Value; NPV: Negative Predictive Value; AUC: Area Under the Curve; CI: Confidence Intervals; NR: Not Reported; PC: Prostate Cancer; ICD: International Classification of Diseases; PSA: Prostate Specific Antigen; GGG: Gleason Grade Group; GS: Gleason Score; CPT: Current Procedural Terminology; HCPCS: Healthcare Common Procedure Coding System; NDC: National Drug Codes; EHR: Electronic Health Records; SEER: Surveillance, Epidemiology and End Results; BM: Bone Metastasis; mPC: Metastatic Prostate Cancer; TNM: Tumor, Node, Metastasis; CCI: Charlson Comorbidity Index; CT: Computed Tomography; MRI: Magnetic Resonance Imaging; PET: Positron Emission Tomography; ML: Machine Learning; XGB: eXtreme Gradient Boosting; DT: Decision Tree; RF: Random Forest; MLP: Multilayer Perceptron; LR: Logistic Regression; NBC: Naïve Bayes Classification; NP: Nomogram Prediction; SLR: Standard Logistic Regression; LRE: Logistic Regression Ensemble

**Supplementary Table 5: Detailed Description of Diagnostic Performance of Advanced Prostate Cancer Phenotypes**

| **Study ID** | **Algorithm Type /Index Test** | **Specifics of an Algorithm / Index Test** | **Reference Standard** | **Sensitivity (95% CI)** | **Specificity (95% CI)** | **PPV (95% CI)** | **NPV (95% CI)** | **Proportion (95% CI) of Patients with an Outcome of Interest** |
| --- | --- | --- | --- | --- | --- | --- | --- | --- |
| **Metastatic Castration-Resistant Prostate Cancer** | | | | | | | | |
| ***Algorithms Based on Claims-Based Codes With or Without Laboratory Values*** | | | | | | | | |
| Freedland et al., 2021 | ICD-9 codes / HCPCS codes/ CPT codes/NDC codes/DRG codes/Lab values | ≥1 evidence of CR defined as: 1) (≥1 ICD-10 code of Z19.2), **OR** 2) (≥1 claim for surgical castration (bilateral orchiectomy, two unilateral orchiectomies) at any time point) AND (≥2 PSA test results after the surgical castration AND ≥1 rise in PSA after nadir, indicating resistance)), **OR** 3) (≥1 claim for surgical castration at any time point AND ≥1 ICD-10 code of R97.21 indicating rising PSA following treatment for malignant neoplasm of prostate, after the surgical castration), **OR** 4) (Medical castration (continuous ADT use for at least 90 days with no gap >30 consecutive days) AND (≥2 PSA test results within the same episode of continuous ADT AND ≥1 rise in PSA after nadir on the same episode of continuous ADT use, indicating resistance)), **OR** 5) (Medical castration AND ≥1 ICD-10 code of R97.21 indicating rising PSA following treatment for PC, during a continuous ADT episode), **OR** 6) Had surgical castration at least 90 days prior to the first observed metastasis diagnosis, **OR** 7) Had continuous ADT throughout 90 days (with no gap >30 days) prior to the first observed metastasis diagnosis, **OR** 8) ≥1 claim for drugs solely used for mCRPC (enzalutamide, DES, polyestrodiol phosphate, estramustine phosphate, pembrolizumab, sipuleucel-T, radium-223, etoposide, carboplatin, cisplatin, cabazitaxel, docetaxel, mitoxantrone) | NR | NR | NR | NR | NR | Optum/MA: 12%; Medicare FFS: 13% |
| Du et al., 2020 | EMR and claims-based variables | Evidence of metastasis between PC diagnosis and CR (defined as having ≥1 of the following after evidence of surgical/medical castration: a) diagnosis of hormone-resistant malignancy status, b) new prescription for antiandrogens, **or** c) PSA level increase after castration (EMR only)) | NR | NR | NR | NR | NR | Optum EMR: 3.1%; Optum claims: 1.6% |
| Thurin et al., 2021 | Database-related variables/ICD codes/drug dispensing /Procedure codes | 1) Identification of date of first metastasis treatment: The first hospital stay with ICD-10 discharge diagnosis codes of C77, C78, C79 for secondary malignant neoplasm, OR denosumab or zoledronic acid dispensing as well as targeted beta or alpha particle therapy and hepatic radiofrequency ablations, OR dispensing of specific mCRPC treatments including docetaxel if preceded by a medical imaging procedure and at least 3 months of continuous ADT, OR RT (non-IMRT, IMRT, or stereotactic radiotherapy), **AND** 2) Identification of CR: Patients on Gonadotropin-releasing hormone analog or surgical castration (orchidectomy, pulpectomy) at the initiation of ADT, DES or degarelix for at least 2 months, OR first dispensing of a CRPC/MCRPC specific treatments within 3 months following the date of diagnosis | Chart review | 77.0% ^Ϣ^ | 100.0% ^Ϣ^ | 92.0% (87.0%-97.0%)^€^; 97.0% (93.0%-100.0%) ^Ϣ^ | 99.0% (98.0%-100.0%)^€^; 99.0 % (97.0%-100.0%) ^Ϣ^ | NR |
|  |  |  |  |  |  |  |  |  |
| **Non-Metastatic Castration-Resistant Prostate Cancer** | | | | | | | | |
| ***Algorithms Based on Claims-Based Codes With or Without Laboratory Values*** | | | | | | | | |
| Du et al., 2020 | EMR and claims-based variables | ≥1 of the following after evidence of surgical/medical castration:  1) diagnosis of hormone-resistant malignancy status,  2) new prescription for antiandrogens, **OR**  3) PSA level increase after castration (EMR only) | NR | NR | NR | NR | NR | Optum EHR: 9.8%; Optum claims: 34.4% |
| Arnold et al., 2019 | Procedure codes/therapy codes/lab value-based codes | An algorithm based on the presence of codes for testosterone suppression therapy, PSA results, and codes indicating the absence of metastatic disease | NR | NR | NR | NR | NR | 3.8% (3.4%-4.0%) |
| Malone et al., 2022 | Procedure codes/therapy codes/lab value-based codes | 1) On ADT or history of surgical castration, 2) Minimum 1 year of ADT use, **AND** 3) PSA <20 ng/ml within 90 days prior to the initiation of ADT | Chart review | 53.30% | 80.3% | 36.2% | 89.1% | NR |
| Malone et al., 2022 | Procedure codes/therapy codes/lab value-based codes | 1) Minimum 1 year of ADT use, **AND** 2) PSA <20 ng/ml within 90 days prior to the initiation of ADT | Chart review | 60.7% | 67.5% | 28.1% | 89.1% | NR |
| Malone et al., 2022 | Procedure codes/therapy codes/lab value-based codes | 1) On ADT or history of surgical castration, **AND** 2) PSA <20 ng/ml within 90 days prior to the initiation of ADT | Chart review | 56.7% | 69.7% | 28.1% | 88.5% | NR |
|  |  |  |  |  |  |  |  |  |
| **Metastatic Castration-Sensitive Prostate Cancer** | | | | | | | | |
| ***Algorithms Based on Claims-Based Codes With or Without Laboratory Values*** | | | | | | | | |
| Freedland et al., 2021 | ICD-9 codes / HCPCS codes/ CPT codes/NDC codes/DRG codes/Lab values | No evidence of CR on or prior to the date of the first observed metastasis diagnosis **AND** evidence of CS defined as: 1) (≥1 ICD-10 code of Z19.1 within 12 months prior to or on the date of the first observed metastasis diagnosis), OR 2) (≥1 claim for surgical castration (bilateral orchiectomy or two unilateral orchiectomies) prior to the date of the first observed metastasis diagnosis) AND (≥2 PSA test results following the surgical castration and within 12 months prior to or on the date of the first observed metastasis diagnosis), OR 3) (Medical castration (continuous use of ADT agents for ≥90 days with no gap in treatment of >30 consecutive days) prior to the date of the first observed metastasis diagnosis) AND (≥2 PSA test results during an episode of ≥90 days of continuous ADT use and within 12 months prior to or on the date of the first observed metastasis diagnosis), OR 4) Hormone/castration naive, defined as no claim for surgical castration prior to the date of the first observed metastasis diagnosis and no claim of ADT in the 18 months prior to the date of the first observed metastasis diagnosis | NR | NR | NR | NR | NR | Optum/MA: 32.0%; Medicare FFS: 22.0% |
| Du et al., 2020 | EMR and claims-based variables | Evidence of metastasis between PC diagnosis and initial indication of ADT | NR | NR | NR | NR | NR | Optum EMR: 1.2%; Optum claims: 1.7% |
|  |  |  |  |  |  |  |  |  |
| **Non-Metastatic Hormone Sensitive Prostate Cancer** | | | | | | | | |
| ***Algorithms Based on Claims-Based Codes and Laboratory Values*** | | | | | | | | |
| Du et al., 2020 | EMR and claims-based variables | Evidence of ADT after new PC diagnosis and no PSA increase (EMR only) | NR | NR | NR | NR | NR | Optum EMR: 3.4%; Optum claims: 30.0% |

^Ϣ^ Adjusted values based on expert feedback

^€^ After weighting values

Abbreviations: NP: Nomogram Prediction; PPV: Positive Predictive Value; NPV: Negative Predictive Value; CI: Confidence Intervals; NR: Not Reported; PC: Prostate Cancer; ICD: International Classification of Diseases; PSA: Prostate Specific Antigen; GGG: Gleason Grade Group; GS: Gleason Score; DRE: Digital Rectal Exam; EMR: Electronic Medical Records; SEER: Surveillance, Epidemiology and End Results; ISUP: International Society of Urological Pathology; MA: Medicare Advantage; FFS: Fee-For-Service; CR: Castration-Resistance; ADT: Androgen Deprivation Therapy; CRPC: Castration-Resistant Prostate Cancer; DES: Di-Ethyl Stilbesterol; ML: Machine Learning; ANN: Artificial Neural Networks; GBDT: Gradient Boosted Decision Tree; LR: Logistic Regression; AJCC: American Joint Committee on Cancer; SD: Standard Deviation; mCRPC: Metastatic Castration Resistant Prostate Cancer; RT: Radiation Therapy; IMRT: Intensity Modulated Radiation Therapy

**Supplementary Table 6: Detailed Description of Diagnostic Performance of Performance Status Phenotype***

| **Author, Year of publication** | **Algorithm type /Index test** | **Specifics of an algorithm / index test** | **Reference standard measure** | **Sensitivity (95% CI)** | **Specificity (95% CI)** | **PPV (95% CI)** | **NPV (95% CI)** | **c-statistic** |
| --- | --- | --- | --- | --- | --- | --- | --- | --- |
| ***Algorithms Based on Claims-Based Codes*** | | | | | | | | |
| Davidoff et al., 2013 | ICD/ HCPCS/ CPT codes | Claims-based predictors including preventive services, evaluation and management visits and other visit types, minor or ambulatory procedures, major procedures, imaging, DME use, and other procedures | Combinations of self-reported functional status, strength, stamina, & exercise, linked to the various functional dimensions and degrees of limitation specified in the ECOG PS scale | 79.0% | 91.7% | 48.3% | 97.8% | 92.0% |
| Sheffield et al., 2018 | ICD/ HCPCS/ CPT/ Standard procedure codes-based prediction model | Predict ECOG PS value (PS-0-1 vs PS>=2) using claims-based indicators as used by Davidoff 2013 and Berenson-Eggers type of services codes including preventive services, evaluation and management visits, and other visit types, minor or ambulatory procedures, major procedures, imaging, DME use, and other; ICD-9 diagnosis for rehabilitation services, HCPCS codes for nursing or personal care services and ICD-9 diagnosis for podiatric care; ICD-9 diagnostic codes for arthritis, diabetes mellitus complications, dementias, difficulty walking, fall, heart failure, lipid abnormality, paralysis, Parkinson's disease, psychiatric illness, sepsis, skin ulcer, stroke/brain injury, vertigo, weakness | ECOG score or Karnofsky PS within 30 days of the initial cancer encounter from EHR | 75.0%^@^ (at predicted probability of 0.078) | 75.0%^@^ | NR | NR | 82.0% |

*focused on the entire cancer population

^@^ at predicted probability of 0.078

Abbreviations: PPV: Positive Predictive Value; NPV: Negative Predictive Value; CI: Confidence Intervals; NR: Not Reported; ICD: International Classification of Diseases; CPT: Current Procedural Terminology; HCPCS: Healthcare Common Procedure Coding System; DME: Durable Medical Equipment; ECOG: Eastern Co-operative Oncology Group; PS: Performance Status; PC: Prostate Cancer; EHR: Electronic Health Records

**Supplementary Table 7: Study Quality Assessment of the Included Studies using QUADAS-2 Tool**

|  | **Risk of Bias** | | | | **Applicability concerns** | | |
| --- | --- | --- | --- | --- | --- | --- | --- |
| **Author, Year of Publication** | **Patient Selection** | **Index Measure** | **Reference Standard** | **Flow and Timing** | **Patient Selection** | **Index Measure** | **Reference Standard** |
| Abdul et al., 2019 | Low | Low | Unclear | Unclear | Low | Low | Unclear |
| Alba et al., 2021 | Low | Low | Low | Low | Low | Low | Low |
| Arnold et al., 2019 | Low | Low | Unclear | Unclear | Low | Low | Unclear |
| Bai et al., 2021 | Low | Low | Low | Low | Low | Low | Low |
| Danciu et al., 2022 | Low | Low | Unclear | Low | Low | Low | Unclear |
| Davidoff et al., 2013 | High | Low | Low | Low | High | Low | Low |
| Dolan et al., 2012 | Low | Low | Low | Low | Low | Low | Low |
| Dong et al., 2022 | Low | Low | Low | Low | Low | Low | Low |
| Du et al., 2020 | Low | Low | Unclear | Unclear | Low | Low | Unclear |
| Ehrenstein et al., 2015 | Low | Low | Low | Low | Low | Low | Low |
| Freedland et al., 2021 | Low | Low | Unclear | Unclear | Low | Low | Unclear |
| Hassett et al., 2014 | Low | Low | Low | Low | Low | Low | Low |
| Hu et al., 2014 | Low | Low | Low | Low | Low | Low | Low |
| Jeong et al., 2012 | Low | Low | Low | Low | Low | Low | Low |
| Leapman et al., 2023 | Unclear | Low | Low | Low | Unclear | Low | Low |
| Liu et al., 2021 | Low | Low | Low | Low | Low | Low | Low |
| Malone et al., 2022 | Low | Unclear | Low | Low | Low | Low | Low |
| Nordstrom et al., 2012 | Low | Low | Low | Low | Low | Low | Low |
| Onukwugha et al., 2014 | Low | Low | Unclear | Low | Low | Low | Low |
| Preisser et al., 2020 | Low | Low | Low | Low | Low | Low | Low |
| Sabbagh et al., 2023 | Low | Low | Unclear | Low | Low | Low | Unclear |
| Sathiakumar et al., 2017 | Low | Low | Low | Low | Low | Low | Low |
| Sheffield et al., 2018 | High | Low | Low | Low | High | Low | Low |
| Shui et al., 2022 | Low | Low | Low | Low | Low | Low | Low |
| Thomsen et al., 2020 | Low | Low | Low | Low | Low | Low | Low |
| Thurin et al., 2021 | Low | Low | Low | Low | Low | Low | Low |
| Vincini et al., 2023 | Low | Low | Unclear | Unclear | Low | Low | Unclear |
| Xiang et al., 2021 | Low | Low | Low | Low | Low | Low | Low |
| Yang et al., 2022 | Low | Low | Low | Low | Low | Low | Low |
